# Seven-Step Total Synthesis of Conidiogenone B Enabled by Radical Cyclizations

**DOI:** 10.1021/jacs.6c02334

**Published:** 2026-04-14

**Authors:** Josephine Bernard, Ziyao Zhang, Mingji Dai

**Affiliations:** † Department of Chemistry, 1371 Emory University, Atlanta, Georgia 30322, United States; 2 Department of Pharmacology and Chemical Biology, School of Medicine, 1371 Emory University, Atlanta, Georgia 30322, United States

## Abstract

We report herein a total synthesis of conidiogenone B, a complex cyclopiane diterpene exhibiting potent antibacterial activity against multidrug-resistant pathogens. Conidiogenone B and its natural congeners feature a congested 6/5/5/5 (A/B/C/D) tetracyclic carbon framework and multiple stereocenters, including four all-carbon quaternary centers. The challenge of conidiogenone total synthesis lies in how to efficiently construct the tetracyclic carbon framework, especially the A/B hydrindane moiety with two vicinal all-carbon quaternary centers at the ring junction. We developed an efficient strategy with a metal-hydride hydrogen atom transfer (MHAT)-initiated reductive olefin-nitrile radical cyclization to close the six-membered A ring and construct the angular methyl containing all-carbon quaternary center adjacent to an existing one, which was formed via a stereoselective Johnson-Claisen rearrangement. Two approaches were investigated to form the B ring. The first one involves a four-step sequence featuring an intramolecular aldol condensation to close the B ring. The second one-step approach utilizes a doubly activated cyclopropane derivative (1-(ethoxycarbonyl)­cyclopropyl)­triphenylphosphonium tetrafluoroborate as a formal 1,3-dipole in a one-pot stepwise (3 + 2) annulation, namely, α-alkylation followed by Wittig olefination, to construct the B ring. In addition, a MHAT-initiated Baran reductive olefin-enone cyclization (BROC) was employed to form the D ring, and a Stork-Danheiser alkylation transposition protocol was used to produce the BROC precursor. Overall, these enabling transformations delivered conidiogenone B in only 7 steps without using any protecting groups by maximizing C–C bond-forming events and minimizing individual functional group manipulation steps to achieve high efficiency and 100% ideality in terms of strategic step count.

## Graphical abstract

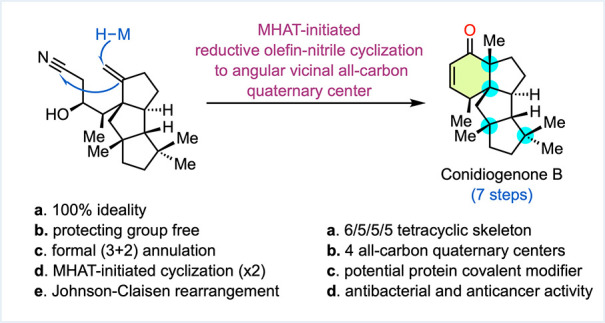

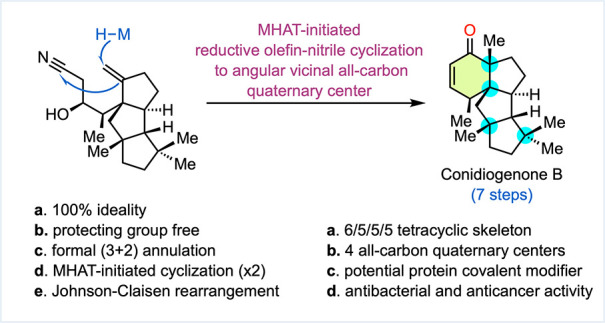

## Introduction

All-carbon quaternary centers are frequently found in natural products and molecules used in medicine. Their existence often helps to enhance the rigidity of the corresponding architecture and improve the related biological activity and metabolic stability.^1^ On the other hand, the existence of all-carbon quaternary centers can significantly increase the synthetic challenge to prepare the target molecule.^2^ Among all-carbon quaternary centers, vicinal quaternary centers are the most difficult ones to install.^3^ Conidiogenone (**1**, Figure 1A) and its natural congeners such as conidiogenones B-G (**2**-**7**), K (**8**), and conidiogenol (**9**) distinguish themselves with four all-carbon quaternary centers including two vicinal all-carbon quaternary centers. The conidiogenones belong to the cyclopiane diterpenoid family and feature a highly congested 6/5/5/5 (A/B/C/D) tetracyclic carbon framework among which exist six or more stereocenters, one linear triquinane, and one angular homo triquinane. Conidiogenone (**1**) and conidiogenol (**9**), first isolated in 2002 by Ugalde and co-workers,^4^ were discovered to exhibit potent activity in inducing conidiogenesis in the fermentation of *Penicillium cyclopium*. Later in 2009, Gu and co-workers isolated conidiogenones B-G (**2**-**7**) from a deep ocean sediment derived fungus *Penicillium* sp. and identified several of them with potent anticancer activity against a panel of cancer cell lines with low micromolar to nanomolar IC_50_ values.^5^ Additionally, conidiogenone B (**2**) and conidiogenol (**9**) showed promising activity against Gram-positive methicillin-resistant *Staphylococcus* aureus (MRSA) and *Staphylococcus epidermidis* and Gram-negative *Pseudomonas aeruginosa* and *Pseudomonas fluorescens* with *MIC* values as low as 8 μg/mL and 16 μg/mL, respectively.^6^ Biosynthetically,^7^ the 6/5/5/5 tetracyclic carbon skeleton is derived from geranylgeranylpyrophosphate (GGPP, **10**, Figure 1B). An enzymatic cationic polyene cyclization would convert **10** to **11** for subsequent alkyl migrations and cyclization to form tricyclic intermediate **12**. A sequence of hydride shift (**12** → **13**), transannular cyclization (**13** → **14**), and carbocation trapping with water (**14** → **15**) would complete the tetracyclic carbon framework to provide deoxyconidiogenol (**15**), a key intermediate to the other conidiogenone family members including **1**-**9** via a series of enzymatic oxidations.

**Figure 1:**
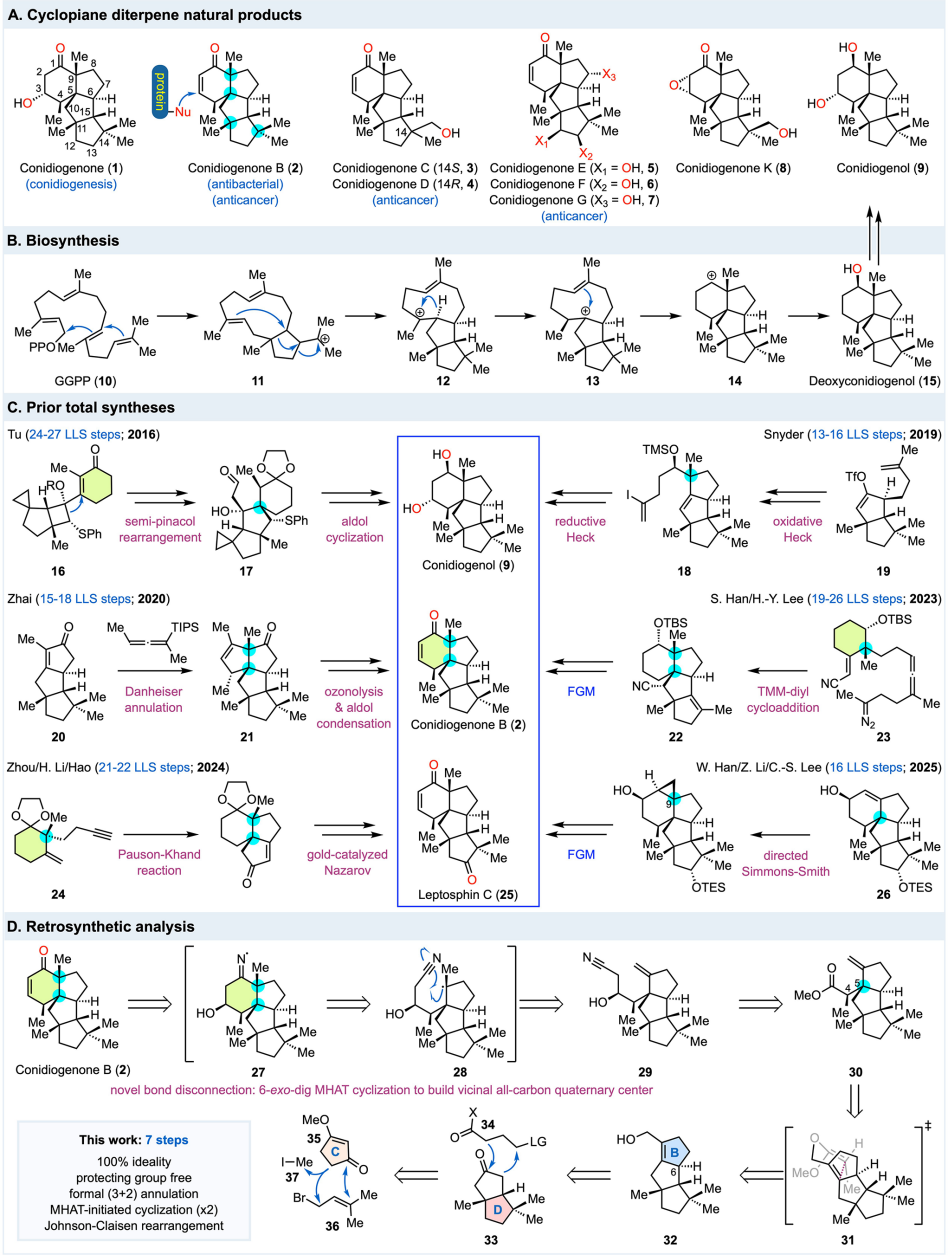
Structures, biosynthesis, prior total syntheses, and retrosynthetic analysis.

The structural complexity and medicinal importance of these cyclopiane diterpenes coupled with their low abundance and isolation burden have generated a significant amount of synthetic attention.^8^ One key problem for their total synthesis is how to efficiently construct its A/B hydrindane ring system with vicinal all-carbon quaternary centers at the ring junction. So far, six elegant total syntheses have been reported (Figure 1C). In 2016, Tu and co-workers reported the first total syntheses of conidiogenone (**1**, 26 steps), conidiogenone B (**2**, 24 steps), and conidiogenol (**9**, 27 steps) and corrected the absolute configuration of **2**.^9^ Their synthesis features a remarkable sulfide-guided semipinacol rearrangement followed by an aldol cyclization to form the vicinal all-carbon quaternary centers of the hydrindane system. In 2019, Snyder and co-workers reported the second total syntheses of conidiogenone (**1**, 15 steps), conidiogenone B (**2**, 13 steps), and conidiogenol (**9**, 16 steps) using an elegant quaternary-center-guided strategy.^10^ For the challenging A/B ring system, they utilized one oxidative Heck reaction and one reductive Heck reaction to build the two all-carbon quaternary centers and close the corresponding five- and six-membered rings, respectively. In 2020, Zhai and co-workers disclosed the third total syntheses of conidiogenone (**1**, 17 steps), conidiogenone B (**2**, 15 steps), and conidiogenol (**9**, 18 steps).^11^ Their synthesis features a Pauson-Khand reaction to build the linear triquinane moiety and a Danheiser annulation to efficiently afford an angular triquinane with vicinal all-carbon quaternary centers, which was then expanded to the angular homotriquinane encoded by the target molecules via a sequence of ozonolysis and aldol condensation. In 2023, S. Han, H.-Y. Lee and co-workers revealed their synthesis of six cyclopiane diterpene natural products including a formal synthesis of conidiogenone B (**2**, 19 steps) and a total synthesis of conidiogenone C (**3**, 26 steps).^12^ Their synthesis starts from Wieland-Miescher ketone and features an efficient trimethylenemethane (TMM) diyl-mediated cycloaddition to forge the linear triquinane on the existing six-membered A ring. In 2024, Zhou, H. Li, Hao and co-workers reported their total syntheses of conidiogenone C (**3**, 21 steps), conidiogenone K (**8**, 22 steps), and 12β-hydroxyconidiogenone C (21 steps).^13^ Their synthesis starts with Wieland-Miescher ketone as well but features a Pauson-Khand reaction followed by a gold-catalyzed Nazarov reaction to build the CD ring system. They also elucidated immunity-related GTPase family M protein 1 (IRGM1) as a potential cellular target of conidiogenone C for its anti-inflammatory activity via covalent modification. Recently, W. Han, Z. Li, C.-S. Lee and co-workers reported an asymmetric total synthesis of leptosphin C (**25**), which contains the same tetracyclic carbon framework as the conidiogenones but was isolated from fungus *Leptosphaeria* sp. XL026. Their synthesis features an asymmetric Robinson annulation to form the A/B hydrindane, a Pauson-Khand reaction to build the C/D ring system, and a directed Simmons-Smith reaction to install the C9 all-carbon quaternary center.^100^ In addition to these total synthesis efforts, Xu and co-workers reported a chemoenzymatic synthesis of the cyclopiane diterpenes by using engineered *E. coli* to produce deoxyconidiogenol (**15**) with the 6/5/5/5 tetracyclic skeleton then chemical methods to advance it to a collection of cyclopiane diterpenes.^14^ These elegant prior arts set a strong foundation for further chemistry innovations in total synthesis of these cyclopiane diterpenes.

Our continued interest^15^ in complex and biologically active natural products which can serve as potential protein covalent modifiers^16^ brought our attention to these cyclopiane diterpenes, especially those containing an α,β-unsaturated enone in the A ring (cf. **2**-**7**). We believe this structural feature would allow them to form a covalent bond with certain cellular proteins containing nucleophilic residues such as cysteine or histidine. Indeed, several cysteine residues were identified to be responsible for the covalent modification of conidiogenone C on IRGM1.^13^ Additionally, the isolation of meleagrin B,^17^ a hybrid natural product of meleagrin and conidiogenone G, indicates that an imidazole-type nucleophile can also add to the β-position of the corresponding cyclohexenone. The key for efficient cyclopiane diterpene total synthesis lies in rapid construction of its highly congested tetracyclic carbon framework especially the A/B hydrindane ring system with vicinal all-carbon quaternary centers at the ring junction. Meanwhile, to maximize synthetic efficiency, step-economy, and ideality, the use of protecting groups and steps for individual functional group manipulation (FGM) such as oxidation state adjustment should be minimized while maximizing the strategic bond forming events.^18^ Recently, we have been leveraging metal-hydride hydrogen atom transfer (MHAT)-initiated Baran reductive olefin cyclization^19^ to synthesize natural products with highly strained and congested ring systems^20^ and wondered about the possibility of using such cyclizations^21^ to build conidiogenone’s tetracyclic carbon framework, especially the A/B ring system with vicinal all-carbon quaternary centers at the ring junction. Retrosynthetically (Figure 1D), we envisioned a MHAT-initiated reductive olefin-nitrile cyclization to build the angular methyl containing all-carbon quaternary center and close the six-membered A ring (**29** → **28** → **27** → **2**).^22^ Such MHAT-initiated reductive olefin-nitrile cyclization has been rarely used in total synthesis^11,22b^ and sometimes fails the challenge of complex natural products.^23^ Furthermore, there has been no report of using it to form an all-carbon quaternary center right next to an existing one. Thus, this strategy, while risky, could help to further test the related methodology and if successful would offer a new solution to the longstanding problem of constructing bicyclic ring systems such as hydrindanes and decalins with vicinal all-carbon quaternary centers at their ring junctions. Nitrile **29** could be derived from γ,δ-unsaturated ester **30** via a 1,2-addition with an acetonitrile derived nucleophile followed by a ketone reduction. Ester **30** could be prepared from allylic alcohol **32** via a Johnson-Claisen rearrangement.^24^ The C6 stereochemistry could control the facial selectivity of the proposed Johnson-Claisen rearrangement, thus directing the stereochemical outcome at C5. A chairlike transition state (**31**) with an *E*-ketene acetal to avoid the steric repulsion between the methyl group and the methylene groups in the B ring would control the formation of the C4 stereochemistry. Allylic alcohol **32** could be derived from **33** via a site selective α-alkylation followed by an intramolecular aldol condensation and oxidation state adjustment. Enantioenriched **33** was prepared in six steps in the Snyder synthesis with a Baran reductive olefin-enone cyclization to close the D ring.^10^ Inspired by the Snyder synthesis, we proposed a modified synthesis of racemic **33** from **35**-**37** via a Stork-Danheiser alkylation transposition protocol^25^ and a slightly different Baran reductive olefin-enone cyclization to close the D ring.^26^

## Results and Discussion

As summarized in Scheme 1, our synthesis commenced with **35**, which was converted to **39** via the Stork-Danheiser alkylation transposition protocol in two steps, namely a one-pot sequential prenylation and methylation to convert **35** to **38** and install the first all-carbon quaternary center and a DIBAL-H reduction followed by HCl hydrolysis to realize the transposition and provide **39**. Enone **39** was then treated with Fe­(acac)_3_ and PhSiH_3_ to trigger the Baran reductive olefin-enone cyclization to close the D ring and afford bicyclic intermediate **33** in 85% yield. With **33** in hand, we moved on to prepare the aldol condensation precursor **42**. Given the low reactivity of alkylating reagents like **34**, we employed a three-step sequence to install the aldehyde side chain,^27^ which started with an α-allylation to convert **33** to **40** followed by a cross-metathesis reaction with acrolein to provide **41**. Subsequent Pd/C-catalyzed selective hydrogenation of the C–C double bond of **41** afforded the aldol condensation precursor **42**. We then explored various base- or acid-promoted aldol condensations to form the B ring. *L*-Proline was first identified as an effective promoter for the aldol cyclization, but the reaction yield was inconsistent. We then discovered that a combination of diisopropylamine and silica could render the cyclization. After a one-pot aldehyde reduction, allylic alcohol **32** with the desired linear triquinane moiety was reliably produced in 50% yield from **41** in two steps. Overall, four steps were needed to advance **33** to **32**, and the aldol condensation step was not amenable to scale-up. To further improve synthetic efficiency and ideality, we wondered about the possibility of a formal (3 + 2) annulation between **33** and (1-(ethoxycarbonyl)­cyclopropyl)­triphenylphosphonium tetrafluoroborate **43**, the Fuchs reagent.^28^ Despite the frequent use of **43** in annulation reactions to form five-membered carbocycles and heterocycles via a tandem sequence of alkylation and Wittig cyclization, most of the nucleophiles involved in the alkylation step to open the doubly activated cyclopropane are derived from 1,3-dicarbonyls, amines, or alcohols,^29^ and enolates derived from monoketone such as **33** are very limited. This seemingly small deviation from the literature precedents turned out not to be a simple task. After extensive investigation, we identified that converting **33** to the corresponding TMS enol ether followed by KO*t*-Bu treatment and trapping the resulting enolate with **43** could give the formal (3 + 2) annulation product **45** in modest yield. One-pot DIBAL-H reduction of the ester group delivered **32** in 21% yield from **33** in two steps. Interestingly, we further discovered that the TMS enol ether formation step was not necessary with NaO*t*-Bu as base, product **32** could be formed in only one step from **33** in 24% yield via a one-pot (3 + 2) annulation and DIBAL-H reduction of a mixed esters of **45** and **46**. This one-step procedure is much more efficient and can be conducted at gram scale.

**Scheme 1:**
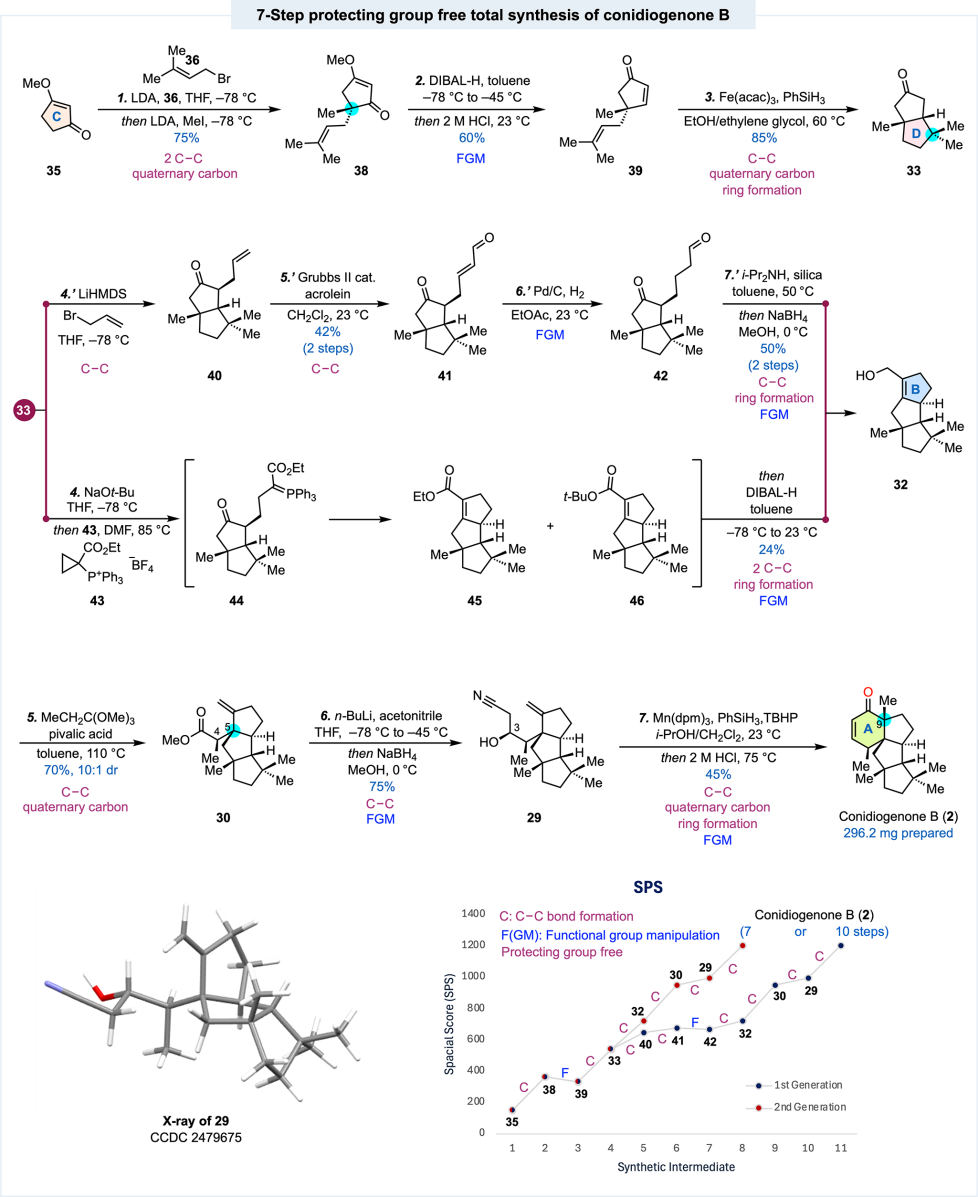
Total Synthesis of (±)-Conidiogenone B

Upon heating **32** with trimethyl orthopropionate in toluene with a catalytic amount of pivalic acid, the Johnson-Claisen rearrangement occurred smoothly and, to our delight, desired product **30** was produced as the major product (70% yield, dr = 10:1) as we predicted in transition state **31** (Figure 1). This rearrangement set up the C4 and C5 stereocenters. It also introduced a carboxylate group as a handle for the subsequent nucleophilic addition with lithio acetonitrile generated by treating acetonitrile with *n*-BuLi. After carefully quenching the reaction with MeOH, NaBH_4_ was added to reduce the resulting ketone to a secondary alcohol in a one-pot treatment, which delivered **29** in 75% yield. The stereochemistry of the C3 secondary alcohol was unambiguously assigned by X-ray crystallographic analysis (CCDC 2479675), which also further validated the C4 and C5 stereochemical outcome of the Johnson-Claisen rearrangement.

With **29** in hand, we started to investigate the MHAT-initiated reductive olefin-nitrile cyclization to close the six-membered ring and build the angular methyl-containing all-carbon quaternary center. We first explored the combination of Fe­(acac)_3_ and PhSiH_3_ in a mixed solvents of EtOH and HFIP at elevated temperatures, the conditions used by Turner, Murphy, Talbot, and co-workers.^22c^ While these conditions worked in simple model substrates to form hydrindane ring systems, they failed on substrate **29** or the corresponding C3 ketone substrate. The main products were characterized as the ones derived from the *exo*-methylene reduction, indicating that the hydrogen atom transfer from the iron center to the olefin did occur, but the radical cyclization on the nitrile did not. Varying the identity of the silane sources and changing the ligands on the iron center did not improve the results. Inspired by Ma’s navirine C total synthesis,^22b^ an elegant application of MHAT-initiated reductive olefin-nitrile cyclization in total synthesis, we turned our attention to Shenvi’s MHAT conditions (Mn­(dpm)_3_/PhSiH_3_/TBHP)^30^ which were used in Ma’s navirine C total synthesis to cyclize an internal olefin with a nitrile to form a five-membered ring. To our delight, under Shenvi’s conditions, desired cyclization occurred to close a six-membered ring in our case and build an all-carbon quaternary center right next to an existing one. This cyclization completed the entire carbon framework of the conidiogenones. After a one-pot acid-promoted imine hydrolysis and alcohol dehydration, (±)-conidiogenone B (296.2 mg) was produced in 45% yield from **29.**

## Conclusions

In summary, starting from monocyclic starting material **35**, a 7-step total synthesis of (±)-conidiogenone B was accomplished. Our synthesis centers on a late-stage MHAT-initiated reductive olefin-nitrile cyclization to forge the six-membered A ring by forming an angular methyl-containing all-carbon quaternary center and the desired ketone functionality and a formal (3 + 2) annulation using the Fuchs reagent to rapidly build the B ring. Other key steps in our total synthesis include a Johnson-Claisen rearrangement to set two adjacent stereocenters including one all-carbon quaternary center, an alternative aldol condensation to build the B ring (for the 10-step approach), and a MHAT-initiated Baran reductive olefin-enone cyclization to close the D ring. These enabling C–C bond forming reactions significantly enhanced overall synthetic efficiency by rapidly increasing structural complexity as shown in the spacial score (SPS) plot (Scheme 1). For the 7-step approach, only one step (step #2) is solely responsible for functional group manipulation (FGM) to reduce the vinylogous ester. This step however qualifies as a strategic redox reaction^18c^ because it enables the use of vinylogous ester **35** as the starting material and releases the enone functionality for the subsequent Baran reductive olefin-enone cyclization, which renders the 7-step synthesis 100% ideality in terms of strategic step count. The other three FGM steps are realized in combination with one or two C–C bond forming events in one-pot manners without reducing the reaction yield. The superior functional group tolerance of the reductive olefin cyclization steps helps to avoid the use of any protecting groups. Furthermore, this synthesis represents a rare application of MHAT-initiated reductive olefin-nitrile cyclization to build fused bicyclic ring systems with vicinal all-carbon quaternary centers at their ring junction by forming one of the all-carbon quaternary centers. Given the novelty of this retrosynthetic bond disconnection logic and the prevalence of such fused ring systems in natural products, this strategy is expected to find broad application in complex natural product total synthesis. This synthesis also presents a rare formal (3 + 2) annulation of the Fuchs reagent with an enolate not derived from a 1,3-dicarbonyl system. Application of the above radical cyclization and formal (3 + 2) annulation strategies to synthesize strained and congested natural products will be further explored and reported in due course.

## Supplementary Material



## Notes

Experimental procedures and NMR spectra for all new compounds (PDF)

## Notes

This paper is dedicated to Professor Samuel J. Danishefsky on the occasion of his 90th birthday.

## Abbreviations

LDA: lithium diisopropylamide
DIBAL-H: diisobutylaluminum hydride
LiHMDS: lithium bis­(trimethylsilyl)­amide
THF: tetrahydrofuran
KO*t*-Bu: potassium *tert*-butoxide
NaO*t*-Bu: sodium *tert*-butoxide
TBHP: *tert*-butyl hydroperoxide
SPS: spacial score
