# Supplementary material for: Seven-Step Total Synthesis of Conidiogenone B Enabled by Radical Cyclizations
Source: J Am Chem Soc. 2026 Apr 14;148(16):17190–6. doi: 10.1021/jacs.6c02334 (PMC13133905; doi:10.1021/jacs.6c02334)

Supporting Information

Seven-Step Total Synthesis of Conidiogenone B Enabled Radical Cyclizations

Josephine Bernard,[1] Ziyao Zhang,[1] Mingji Dai*,[1,2]

[1]Department of Chemistry, Emory University, Atlanta, Georgia 30322, United States.

[2]Department of Pharmacology and Chemical Biology, School of Medicine, Emory University, Atlanta, Georgia 30322, United States.

Table of Content

A. General Methods..........S3

B. Experimental Procedures and Spectra Data..........S4

C. X-ray Crystal Data..........S13

D. NMR Comparison Tables..........S14

E. References..........S16

F. ^{1}H and ^{13}C NMR Spectra..........S17

A. General Methods.

All commercially available compounds were purchased from Sigma-Aldrich, Alfa-Aesar, Oakwood chemicals, Ambeed, and ChemScene unless otherwise noted. Materials obtained from commercial suppliers were used without further purification. NMR spectra were recorded on Bruker spectrometers (^{1}H at 400 MHz, 600 MHz, 800 MHz and ^{13}C at 100 MHz, 150 MHz, 200 MHz). Chemical shifts (δ) were given in ppm with reference to solvent signals [^{1}H NMR: $CDCl_3$ (7.26); ^{13}C NMR: $CDCl_3$ (77.16)]. ^{1}H NMR data are reported as follows: chemical shift (δ ppm), multiplicity (s = singlet, d = doublet, t = triplet, q = quartet, m = multiplet, br = broad, app = apparent), coupling constant (Hz), and integration. IR spectra were collected on a Nicolet iS10 FT-IR spectrometer. Mass spectra were taken on a Thermo Finnigan LTQ-FTMS spectrometer with APCI, ESI. Column chromatography was performed on silica gel. All reactions sensitive to air or moisture were conducted under argon atmosphere in dry solvents under anhydrous conditions, unless otherwise noted. Dry THF, CH_2Cl_2 and toluene were processed via PureProcessTechnology GS-SPS-5-CM system. Dry acetonitrile and DMF were purchased from Sigma-Aldrich. All other solvents and reagents were used as obtained from commercial sources without further purification. Room (ambient) temperature (r.t.) is around 23 °C.

B. Experimental Procedures and Spectra Data

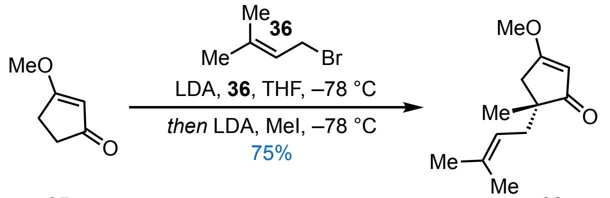

Compound 38: To a flame-dried 500 mL round-bottom flask was added diisopropylamine (10.5 mL, 74.6 mmol, 1.1 equiv) and THF (150 mL). The mixture was cooled to –78 °C, and *n*-BuLi (2.5 M, 29.8 mL, 74.6 mmol, 1.1 equiv) was added dropwise. The mixture was stirred at –78 °C for 1 hour. A solution of 3-methoxy-2-cyclopenten-1-one **35** (7.6 g, 67.9 mmol, 1.0 equiv) in THF (10 mL) was introduced dropwise over 30 min. After stirring for 1 hour at –78 °C, 1-bromo-3-methylbut-2-ene (12.0 g, 81.4 mmol, 1.2 equiv) was added dropwise, and the reaction was maintained at –78 °C for 1 hour before warming to ambient temperature. The mixture was cooled again to –78 °C, and a second portion of LDA (ca. 1.0 M in THF, 74.6 mmol, 1.1 equiv, prepared in another flask) was added dropwise. After stirring for 1 hour at –78 °C, iodomethane (5.1 mL, 81.4 mmol, 1.2 equiv) was added dropwise, and the reaction was stirred for an additional 1 hour at –78 °C before warming to ambient temperature. The reaction was quenched with saturated aqueous NH_4Cl (100 mL) and extracted with EtOAc (3 × 200 mL). The combined organic layers were washed sequentially with 2 M aqueous HCl (100 mL), saturated aqueous $NaHCO_3$ (100 mL), and brine (100 mL), dried over Na_2SO_4, filtered, and concentrated under reduced pressure. Purification by flash column chromatography on silica gel (15% to 30% EtOAc in hexanes) afforded **38** (9.8 g, 59.5 mmol, 75% yield) as a pale-yellow oil.

Rf: 0.4 in 1:1 hexane to ethyl acetate.

^{1}H NMR (400 MHz, $CDCl_3$) δ 5.22 (t, *J* = 1.1 Hz, 1H), 5.00 (m, 1H), 3.83 (s, 3H), 2.53 (dd, *J* = 17.6, 1.2 Hz, 1H), 2.25 (dd, *J* = 17.6, 1.1 Hz, 1H), 2.17 (dt, *J* = 7.9, 1.1 Hz, 2H), 1.68 (d, *J* = 1.3 Hz, 3H), 1.61 (d, *J* = 1.3 Hz, 3H), 1.14 (s, 3H).

^{13}C NMR (101 MHz, $CDCl_3$) δ 210.6, 188.9, 134.7, 119.6, 102.6, 58.6, 48.1, 41.1, 36.1, 26.1, 24.1, 18.1.

IR (neat): 2966, 2925, 2866, 2225, 1691, 1591, 1375, 1349, 1266, 994 cm^{-1}.

HRMS m/z (APCI): calc. for $C_{12}H_{19}O_2$ $[M+H]^+$: 195.13796, found: 195.13836.

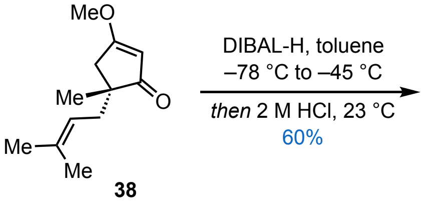

Compound 39[1]**:** To a solution of **38** (16.0 g, 82.4 mmol, 1.0 equiv) in toluene (300 mL) at –78 °C was added dropwise diisobutylaluminum hydride (DIBAL-H, 25% wt in toluene, 66 mL, 99.0 mmol, 1.2 equiv) over 30 min. The reaction mixture was then warmed to –45 °C and stirred for 1 hour, or until TLC indicated complete consumption of the starting material. A solution of 2.0 M HCl in MeOH (1:2, 200 mL) was added, and the mixture was stirred at 23 °C overnight. The reaction mixture was extracted with EtOAc (3 × 100 mL). The combined organic layers were dried over Na_2SO_4, filtered, and concentrated under reduced pressure. Purification by flash column chromatography on silica gel (2% EtOAc in hexanes) afforded enone **39** (8.0 g, 48.7 mmol, 60% yield) as a yellow solid.

Rf: 0.5 in a 5:1 of hexane to ethyl acetate.
^{1}H NMR (400 MHz, $CDCl_3$) δ 7.44 (d, *J* = 5.6 Hz, 1H), 6.04 (d, *J* = 5.6 Hz, 1H), 5.04 (m, 1H), 2.29 (d, *J* = 18.6 Hz, 1H), 2.26 – 2.12 (m, 2H), 2.09 (d, *J* = 18.6 Hz, 1H), 1.70 (d, *J* = 1.2 Hz, 3H), 1.59 (d, *J* = 1.4 Hz, 3H), 1.21 (s, 3H).
^{13}C NMR (101 MHz, $CDCl_3$) δ 210.3, 173.0, 135.2, 132.1, 119.4, 47.6, 45.7, 38.8, 26.2, 26.1, 18.1.
IR (neat): 3014, 2969, 2927, 2869, 2361, 2340, 1737, 1716, 1365, 1228 cm^{-1}.
HRMS m/z (APCI): calc. for $C_{11}H_{17}O$ $[M+H]^+$: 165.12739, found: 165.12759.

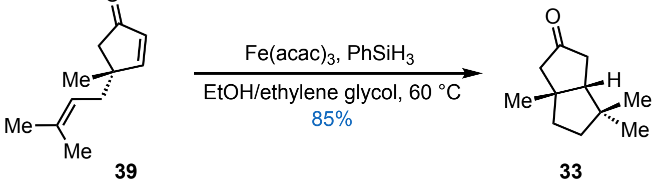

Compound 33[2]**.** The reaction was carried out in 4 parallel reactions. To a solution of enone **39** (2.0 g, 12.2 mmol, 1.0 equiv) and $Fe(acac)_3$ (862 mg, 2.44 mmol, 0.2 equiv) in ethylene glycol (10 mL) and ethanol (50 mL) was added phenylsilane (3.76 mL, 30.4 mmol, 5.0 equiv). The reaction mixture was heated to 60 °C and stirred for 4 hours, then cooled to ambient temperature. The 4 parallel reaction mixtures were combined and diluted with H_2O (50 mL). The mixture was directly rotavaped until all the alcohols were separated and collected in a receiving flask. The water phase was extracted with EtOAc (2 × 100 mL). The alcohol phase containing some desired product was added equal amount of water and extracted with hexane (3 × 100 mL).

The combined organic layers were washed with brine (50 mL), dried over Na_2SO_4, filtered, and concentrated under reduced pressure. Purification by flash column chromatography on silica gel (2–5% EtOAc in hexanes) afforded ketone **33** (6.8 g, 41.5 mmol, 85% yield) as a colorless oil.
Note: It's important to collect the alcohols and do additional extractions. Otherwise, part of the product will be lost.

Rf: 0.55 in 5:1 of hexane to ethyl acetate.
^{1}H NMR (400 MHz, $CDCl_3$) δ 2.38 (ddd, *J* = 19.3, 9.9, 1.6 Hz, 1H), 2.30 – 2.25 (m, 1H), 2.22 (dd, *J* = 15.8, 1.5 Hz, 1H), 2.17 (d, *J* = 1.4 Hz, 1H), 1.86 (dd, *J* = 9.9, 3.2 Hz, 1H), 1.80 – 1.65 (m, 2H), 1.58 – 1.54 (m, 2H), 1.22 (s, 3H), 1.04 (s, 3H), 0.83 (s, 3H).
^{13}C NMR (101 MHz, $CDCl_3$) δ 221.1, 57.6, 53.2, 47.1, 42.0, 41.3, 40.2, 39.8, 30.7, 30.5, 24.5.
IR (neat): 2951, 2926, 2869, 1739, 1465, 1384, 1366, 1167, 1106, 921 cm^{-1}.
HRMS m/z (APCI): calc. for $C_{11}H_{19}O$ [M+H]$^+$: 167.14304, found: 167.14345.

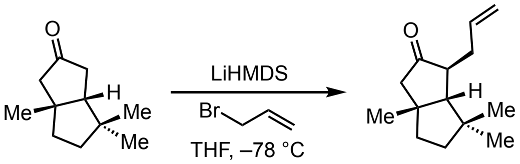

33 **40**

Compound 40[3]**.** To a flame-dried 100 mL round-bottom flask under argon was added ketone **33** (6.8 g, 48.4 mmol, 1.0 equiv) in dry THF (ca. 200 mL). The solution was cooled to –78 °C, and lithium bis(trimethylsilyl)amide (1.0 M in THF, 53.2 mL, 53.2 mmol, 1.1 equiv) was added dropwise over 10 min. The reaction mixture was stirred at –78 °C for 1.5 hours before allyl bromide (4.6 mL, 53.2 mmol, 1.2 equiv) was added dropwise. The mixture was maintained at –78 °C and then allowed to warm gradually to ambient temperature over 8 hours. The reaction was quenched with saturated aqueous NH_4Cl (10 mL) and extracted with EtOAc (2 × 100 mL). The combined organic layers were washed with brine (100 mL), dried over Na_2SO_4, filtered, and concentrated under reduced pressure. Crude alkene **40** (9.0 g, dark brown oil) was directly used without further purification.

Rf: 0.6 in 5:1 of hexane to ethyl acetate.
^{1}H NMR (400 MHz, $CDCl_3$) δ 5.79 – 5.64 (m, 1H), 5.06 (d, *J* = 2.2 Hz, 1H), 5.03 (d, *J* = 1.6 Hz, 1H), 2.48 – 2.39 (m, 1H), 2.38 – 2.26 (m, 2H), 2.23 – 2.05 (m, 2H), 1.75 – 1.62 (m, 3H), 1.59 – 1.52 (m, 2H), 1.17 (s, 3H), 1.04 (s, 3H), 0.91 (s, 3H).

^{13}C NMR (101 MHz, CDCl$_3$) δ 221.0, 136.2, 117.1, 62.2, 53.5, 50.4, 44.9, 42.2, 41.4, 40.0, 36.4, 31.4, 30.4, 25.1.

IR (neat): 2949, 2864, 2359, 2341, 1700, 1497, 1410, 1264, 1132, 910 cm^{-1}.

HRMS m/z (APCI): calc. for $C_{14}H_{23}O$ [M+H]+: 207.17434, found: 207.17454.

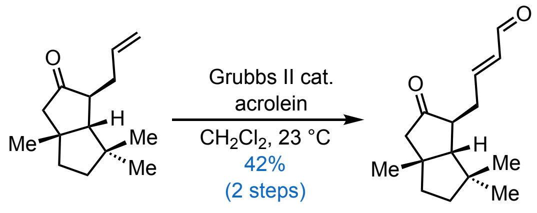

40 41

Compound 41[4]**.** The reaction was carried out in 9 parallel reactions. To a flame-dried 250 mL flask under argon was added crude alkene **40** (1.0 g, 4.9 mmol, 1.0 equiv) in CH_2Cl_2 (20 mL). Acrolein (1.6 mL, 24.3 mmol, 5.0 equiv) was added, followed by Grubbs second-generation catalyst (83 mg, 0.098 mmol, 0.02 equiv). The reaction mixture was stirred at 40 °C for 4 hours. The 9 parallel reactions were combined. The solvent was removed under reduced pressure, and the crude residue was purified by flash column chromatography on silica gel (5–10% EtOAc in hexanes) to afford **41** (4.1 g, 17.5 mmol, 42% yield over two steps) as a pale-yellow oil.

Rf: 0.4 in 4:1 of hexane to ethyl acetate.

^{1}H NMR (400 MHz, CDCl$_3$) δ 9.51 (d, J = 7.8 Hz, 1H), 6.81 (dt, J = 15.7, 7.1 Hz, 1H), 6.13 (ddt, J = 15.6, 7.9, 1.4 Hz, 1H), 2.72 – 2.62 (m, 1H), 2.48 – 2.36 (m, 3H), 2.19 (d, J = 17.8 Hz, 1H), 1.79 – 1.66 (m, 2H), 1.59 (t, J = 6.5 Hz, 2H), 1.52 (d, J = 5.8 Hz, 1H), 1.16 (s, 3H), 1.05 (s, 3H), 0.95 (s, 3H).

^{13}C NMR (101 MHz, CDCl$_3$) δ 218.8, 193.8, 155.7, 134.8, 62.6, 53.1, 49.5, 44.8, 42.2, 41.1, 39.7, 34.7, 31.4, 30.5, 25.1.

IR (neat): 2951, 2868, 1733, 1692, 1457, 1407, 1375, 1292, 1269, 1246 cm^{-1}.

HRMS m/z (APCI): calc. for $C_{15}H_{23}O_2$ [M+H]$^+$: 235.16926, found: 235.16929.

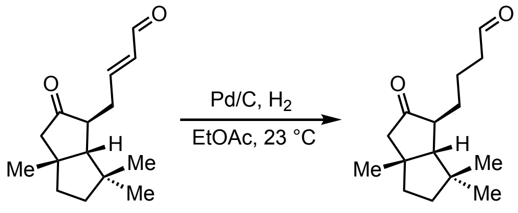

41 42

Compound 42[4]**.** To a 100 mL round-bottom flask under argon was added **41** (4.1 g, 17.5 mmol, 1.0 equiv), 10% Pd/C (205 mg, 5% wt), and EtOAc (200 mL, 0.2 M). The flask was evacuated and backfilled 6 times with hydrogen (balloon), and the mixture was stirred at ambient temperature for 30 min. Upon completion as determined by TLC, the reaction mixture was filtered through a pad of Celite and washed with additional EtOAc. The combined filtrate was concentrated under reduced pressure to afford aldehyde **42** (4.1 g) as a crude product, which was used directly in the subsequent step without further purification.

Rf: 0.4 in 4:1 of hexane to ethyl acetate.
^{1}H NMR (600 MHz, $CDCl_3$) δ 9.77 (t, *J* = 1.6 Hz, 1H), 2.48 – 2.44 (m, 2H), 2.33 (dd, *J* = 18.2, 1.8 Hz, 1H), 2.21 – 2.17 (m, 2H), 1.78 – 1.63 (m, 5H), 1.59 – 1.55 (m, 4H), 1.18 (s, 3H), 1.08 (s, 3H), 0.91 (s, 3H).
^{13}C NMR (101 MHz, $CDCl_3$) δ 221.3, 202.3, 63.5, 53.4, 50.5, 45.0, 44.0, 42.3, 41.3, 40.0, 31.9, 31.6, 30.5, 24.9, 20.3.
IR (neat): 2950, 2867, 1730, 1700, 1460, 1408, 1386, 1375, 1096, 1064, 997 cm^{-1}.
HRMS m/z (APCI): calc. for $C_{15}H_{25}O_2$ $[M+H]^+$: 237.18491, found: 237.18479.

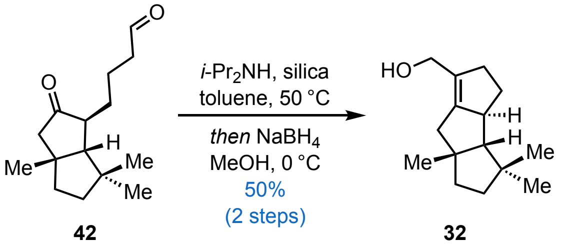

Compound 32. This reaction is carried out in 60 parallel reactions. To a flame-dried 20 mL microwave flask was added aldehyde **42** (50 mg, 0.21 mmol, 1.0 equiv) and toluene (8 mL). Silica (70 mg, 0.212 mmol, 0.5 equiv) was added. The mixture was stirred for 5 min. Diisopropylamine (28 mg, 0.28 mmol, 1.3 equiv) was added. The reaction mixture was stirred at 50 °C (sand bath; 500 mL bath filled with sand, surface temperature 43 °C, bottom temperature 56 °C) for 72 hours. All the reactions were combined and cooled in an ice bath. MeOH (100 mL) was added, and the solution was stirred for 10 min at 0 °C before $NaBH_4$ (970 mg, 25.2 mmol, 2.0 equiv) was added in portions. The mixture was stirred for an additional 30 min at 0 °C and quenched with water (100 mL). The aqueous phase was extracted with EtOAc (3 × 100 mL), and the combined organic layers were dried over Na_2SO_4, filtered, and concentrated under reduced pressure. Purification by flash column chromatography on silica gel (10%-20% EtOAc in hexanes) afforded allylic alcohol **32** (1.4 g, 6.4 mmol, 50% yield over two steps from **41**) as a colorless oil.

Rf: 0.5 in 3:1 of hexane to ethyl acetate.

^{1}H NMR (400 MHz, CDCl$_3$) δ 4.15 (m, 2H), 2.84 (m, 1H), 2.70 – 2.50 (m, 2H), 2.17 – 2.09 (m, 2H), 1.94 (d, J = 12.9 Hz, 1H), 1.62 – 1.57 (m, 3H), 1.45 – 1.33 (m, 3H), 1.12 (d, J = 0.5 Hz, 3H), 1.10 – 1.06 (m, 1H), 0.98 (s, 2x3H).

^{13}C NMR (101 MHz, CDCl$_3$) δ 148.8, 129.2, 69.2, 60.4, 54.9, 54.4, 40.8, 40.5, 40.4, 39.9, 37.6, 32.8, 31.9, 29.9, 25.6.

IR (neat): 3383, 2949, 2929, 2864, 1458, 1260,1093, 1025, 779, 723 cm^{-1}.

HRMS m/z (APCI): calc. for $C_{15}H_{25}O$ $[M+H]^+$: 221.18999, found: 221.19031.

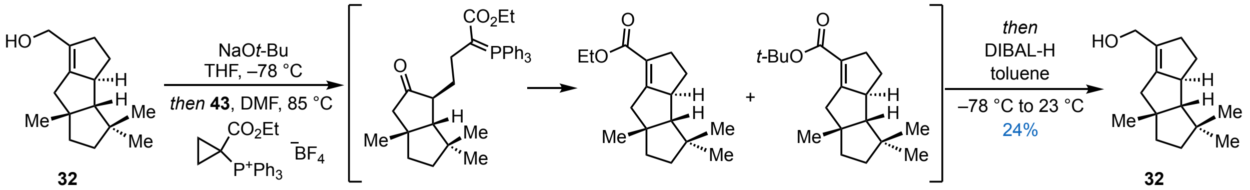

32 **43** **44** **45** **46** **32**

Formal (3+2) annulation route to compound 45, 46 and 32. To a flame dried 100 mL round bottom flask was added ketone **33** (1.0 g, 6.0 mmol, 1.0 equiv) and THF (30 mL, 0.2 M). The mixture was cooled to –78 °C and a solution of NaO*t*-Bu (1.2 g, 12.0 mmol, 2.0 equiv) in THF (15 mL) was added dropwise. The mixture was kept stirring at –78 °C for 30 min and a solution of Fuchs reagent **43** (5.8 g, 12.6 mmol, 2.1 equiv) in DMF (15 mL) was added dropwise. The reaction was warmed up to room temperature in 30 min, then heated to 85 °C for another 36 h. Upon finishing, the reaction was again cooled down to –78 °C, and DIBAL-H (ca. 1 M in toluene, 120 mL, 120 mmol, 20.0 equiv) was added dropwise. The reaction was kept stirring at –78 °C for 2 h, then warmed up to room temperature and kept stirring for 1 h. The reaction was quenched with Rochelle's salt and stirred overnight until two phases were clear. The mixture was extracted with EtOAc (3 × 100 mL). The combined organic layer was washed with brine, dried over Na_2SO_4 and concentrated. Purification by flash column chromatography on silica gel (10% EtOAc in hexanes) afforded allylic alcohol **32** (320 mg, 1.45 mmol, 24% yield) as a colorless oil. The spectra data are the same as the ones obtained from the aldol condensation approach.

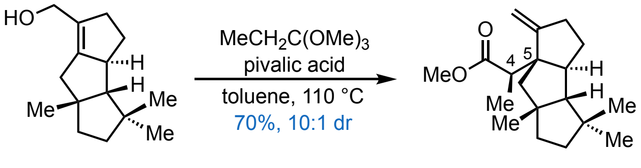

32 **30**

Compound 30[5]**.** The reaction was carried in 14 parallel reactions. To a 20 mL microwave flask was added allylic alcohol **32** (100 mg, 0.454 mmol, 1.0 equiv), pivalic acid (13.9 mg, 0.136 mmol, 0.3 equiv), and

trimethyl orthopropionate (368 μL, 2.27 mmol, 5.0 equiv) in toluene (10 mL). The reaction mixture was stirred at 110 °C for 8 hours under argon. After cooling to ambient temperature, all the reactions were combined and concentrated under reduced pressure. The residue was purified by flash column chromatography on silica gel (2% to 5% EtOAc in hexane) to afford **30** as a mixture of two inseparable diastereomers (1.3 g, 4.5 mmol, 70% yield, 10:1 dr) as a colorless oil.

Rf: 0.85 in 5:1 of hexane to ethyl acetate

^{1}H NMR (800 MHz, $CDCl_3$) δ 5.01 – 5.00 (m, 1H), 4.81 (t, *J* = 2.6 Hz, 1H), 3.60 (s, 3H), 2.60 – 2.47 (m, 2H), 2.42 (q, *J* = 7.1 Hz, 1H), 2.37 (dd, *J* = 8.2, 6.4 Hz, 1H), 1.93 (d, *J* = 13.5 Hz, 1H), 1.78 (d, *J* = 13.5 Hz, 1H), 1.65 (dtd, *J* = 13.0, 10.0, 6.4 Hz, 1H), 1.49 – 1.43 (m, 4H), 1.31 (tdd, *J* = 5.8, 3.1, 1.3 Hz, 1H), 1.18 (dd, *J* = 8.2, 1.2 Hz, 1H), 1.15 (s, 3H), 1.11 (d, *J* = 7.1 Hz, 3H), 1.00 (s, 3H), 0.94 (s, 3H).

^{13}C NMR (201 MHz, $CDCl_3$) δ 176.4, 157.6, 107.8, 68.1, 60.6, 51.9, 51.3, 50.6, 50.6, 47.3, 42.4, 42.1, 40.1, 32.8, 31.2, 30.1, 28.5, 25.7, 13.9.

IR (neat): 2947, 2931, 2867,1736, 1458, 922, 911, 829 cm^{-1}.

HRMS m/z (APCI): calc. for $C_{19}H_{31}O_2$ [M+H]$^+$: 291.23186, found: 291.23062.

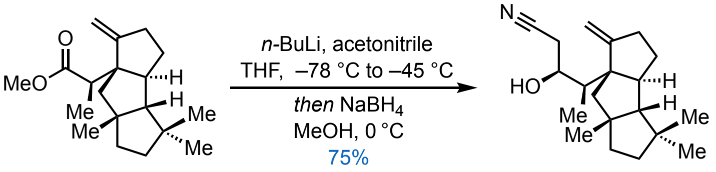

30 **29**

Compound 29. A solution of acetonitrile (475 μL, 9.0 mmol, 2.0 equiv) in THF (34 mL) was cooled to –78 °C under argon. Then *n*-butyllithium (2.5 M in hexanes, 3.6 mL, 9 mmol, 2.0 equiv) was added dropwise. The reaction mixture was stirred at –78 °C for 1 hour, after which ester **30** (1.3 g, 4.5 mmol, 1.0 equiv) in THF (0.50 mL) was added dropwise. The mixture was warmed to –45 °C and stirred for 2 hours, then further warmed to 0 °C. MeOH (20 mL) was added, and after 10 min $NaBH_4$ (346.5 mg, 9 mmol, 2.0 equiv) was introduced. The reaction was stirred for an additional 30 min at 0 °C, then quenched with brine (10 mL) and extracted with EtOAc (3 × 5 mL). The combined organic layers were dried over Na_2SO_4, filtered, and concentrated under reduced pressure. Purification by flash column chromatography on silica gel (10% EtOAc in hexanes) afforded **29** (1.0 g, 3.3 mmol, 75% yield, 10:1 dr) as a colorless oil.

Rf: 0.85 in 5:1 of hexane to ethyl acetate.

^{1}H NMR (800 MHz, $CDCl_3$) δ 5.16 (s, 1H), 4.99 (s, 1H), 4.06 (ddd, *J* = 8.3, 5.3, 3.1 Hz, 1H), 2.60 (ddd, *J* = 10.0, 4.7, 2.3 Hz, 2H), 2.48 (dd, *J* = 16.7, 3.1 Hz, 1H), 2.39 (dd, *J* = 16.7, 8.2 Hz, 1H), 2.25 (dd, *J* = 9.0, 6.6 Hz, 1H), 1.82 (d, *J* = 13.1 Hz, 1H), 1.79 – 1.73 (m, 1H), 1.72 (dd, *J* = 7.1, 5.2 Hz, 1H), 1.65 (d, *J* = 13.1

Hz, 1H), 1.56 – 1.44 (m, 6H), 1.34 (dd, J = 9.1, 7.9 Hz, 1H), 1.19 (s, 3H), 0.99 (s, 3H), 0.96 (s, 3H), 0.93 (d, J = 7.1 Hz, 3H).

^{13}C NMR (101 MHz, $CDCl_3$) δ 159.0, 118.7, 109.0, 69.6, 66.9, 61.0, 53.2, 50.8, 47.3, 43.6, 42.3, 42.0, 40.0, 33.2, 30.9, 29.9, 26.7, 25.6, 23.1, 10.2.

IR (neat): 3480, 3069, 2945, 2868, 2364, 1459, 1371, 1309, 1132, 979 cm^{-1}.

HRMS m/z (APCI): calc. for $C_{20}H_{32}ON$ $[M+H]^+$: 302.24784, found: 302.24823.

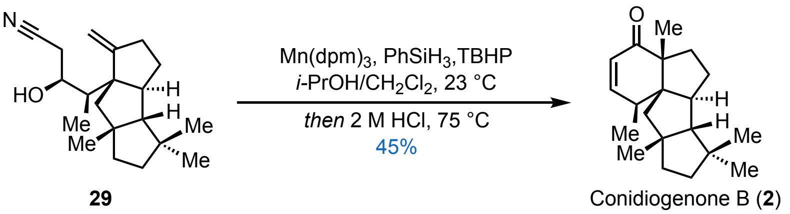

Conidiogenone B (2)[6,7]**.** The reaction was carried out in 14 parallel reactions. Compound **29** (50 mg, 0.17 mmol, 1.0 equiv), $Mn(dpm)_3$ (103.0 mg, 0.17 mmol, 1.0 equiv), phenylsilane (42 µL, 0.34 mmol, 2.0 equiv), and TBHP (22 µL, 70% in H_2O, 0.17 mmol, 1.0 equiv) were dissolved in a 4:1 mixture of *i*-PrOH/CH_2Cl_2 (3.0 mL *i*-PrOH, 0.7 mL CH_2Cl_2) and stirred at 23 °C for 6 hours. Upon completion of the reaction, each mixture was treated with 2 M HCl (2 mL) and heated to 75 °C for 1 hour. The reaction was cooled to ambient temperature and quenched with brine (5 mL). All the parallel mixtures were combined. The water phase was extracted with CH_2Cl_2 (2 × 50 mL). The combined organic layers were dried over Na_2SO_4, filtered, and concentrated under reduced pressure. Purification by flash column chromatography on silica gel (1% to 2% EtOAc in hexanes) afforded conidiogenone B (**2**) (296.2 mg, 1.03 mmol, 45% yield) as a colorless solid.

Rf: 0.52 in 10:1 of hexane to ethyl acetate

^{1}H NMR (800 MHz, $CDCl_3$) δ 6.94 (dd, J = 10.0, 5.9 Hz, 1H), 5.97 (dd, J = 10.0, 1.1 Hz, 1H), 2.68 (p, J = 7.0 Hz, 1H), 2.25 (q, J = 5.4 Hz, 1H), 2.11 – 2.06 (m, 1H), 1.99 (d, J = 14.6 Hz, 1H), 1.71 – 1.56 (m, 7H), 1.53 – 1.47 (m, 1H), 1.39 (d, J = 5.3 Hz, 1H), 1.28 (s, 2H), 1.20 (d, J = 7.3 Hz, 3H), 1.20 – 1.18 (m, 1H), 1.18 (s, 3H), 1.06 (s, 3H), 0.96 (s, 3H).

^{13}C NMR (151 MHz, $CDCl_3$) δ 206.2, 154.6, 127.6, 73.8, 59.1, 57.6, 56.2, 52.4, 48.1, 42.9, 40.9, 39.2, 39.1, 37.7, 34.5, 32.7, 31.1, 27.3, 21.1, 18.9.

IR (neat): 2967, 2888, 1721, 1654, 1461, 1374, 1292, 1182, 1093, 978, 721 cm-1.

HRMS m/z (APCI): calc. for $C_{20}H_{31}O$ [M+H]+: 287.23694, found: 287.23653.

C. X-Ray Crystal Data

Crystal Data and Experimental for **29**

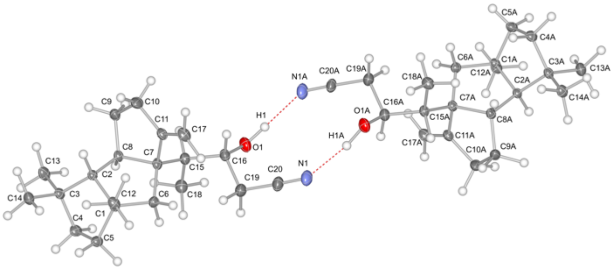

Experimental. Single colorless prism-shaped crystals of **29** recrystallized from DCM by slow evaporation. A suitable crystal with dimensions 0.25 × 0.22 × 0.08 mm^3 was selected and mounted on a loop with paratone on a XtaLAB Synergy, Dualflex, HyPix diffractometer. The crystal was kept at a steady T = 100.0(1) K during data collection. The structure was solved with the ShelXT (Sheldrick, 2015) solution program and by using Olex2 1.5-alpha (Dolomanov et al., 2009) as the graphical interface. The model was refined with olex2.refine 1.5-alpha (Bourhis et al., 2015) using full matrix least squares minimisation on $\boldsymbol{F^2}$.

Crystal Data. $C_{20}H_{31}NO$, M_r = 301.475, orthorhombic, $Pna2_1$ (No. 33), a = 14.7671(3) Å, b = 7.9443(2) Å, c = 29.8539(5) Å, a = 90°, b = 90°, g = 90°, V =3502.29(13) $Å^3$, T = 100.0(1) K, Z = 8, Z' = 2, m(Cu Ka) = 0.524, 51438 reflections measured, 7114 unique (R_{int} = 0.0419) which were used in all calculations. The final wR_2 was 0.0794 (all data) and $R1$ was 0.297 (I≥2 s(I)).

**Compound**	**29**
CCDC2479675	
Formula	$C_{20}H_{31}NO$
D_{calc}/ g cm^{-3}	1.144
m/mm^{-1}	0.524
Formula Weight	301.475
Color	colorless
Shape	prism
Size/mm^3	0.25 × 0.22 × 0.08
T/K	100.0(1)
Crystal System	orthorhombic
Flack Parameter	-0.04(7)
Hooft Parameter	-0.04(7)
Space Group	$Pna2_1$
a/Å	14.7671(3)
b/Å	7.9443(2)
c/Å	29.8539(5)
$a/^{\circ}$	90
$b/^{\circ}$	90
$g/^{\circ}$	90
V/$Å^3$	3502.29(13)
Z	8
Z'	2
Wavelength/Å	1.54184
Radiation type	Cu K_{α}
$Q_{min}/^{\circ}$	2.96
$Q_{max}/^{\circ}$	77.08
Measured Refl's	51438
Indep't Refl's	7114
Refl's I ≥ 2 s(I)	6913
R_{int}	0.0419
Parameters	848
Restraints	1021
Largest Peak	0.531
Deepest Hole	-0.243
GooF	1.0726
wR_2 (all data)	0.0794
wR_2	0.0789
R_1 (all data)	0.0306
R_1	0.297

D. NMR Comparison Tables

Table S1. ^{1}H NMR ($CDCl_3$) Spectroscopic Comparison of Zhai's,[8] Snyder's,[9] Tu's,[10] and natural isolation[11] to our syntheses of Conidiogenone B

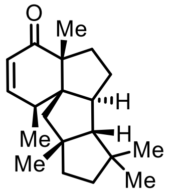

Conidiogenone B

No.	Our Sample (800 MHz)	Zhai's sample (500 MHz)	Snyder's sample (500 MHz)	Tu's sample (400 MHz)	Natural (500 MHz)
1	6.94 (dd, J = 10.0, 5.9 Hz, 1H)	6.94 (dd, J = 10.0, 5.9 Hz, 1H)	6.93 (dd, J = 10.0, 5.9 Hz, 1H)	6.95 (dd, J = 10.0, 6.0 Hz, 1H)	6.93 (dd, J = 9.9, 6.1 Hz, 1H)
2	5.97 (dd, J = 10.0, 1.1 Hz, 1H)	5.97 (dd, J = 10.0, 1.1 Hz, 1H)	5.96 (dd, J = 10.0, 1.1 Hz, 1H)	5.97 (dd, J = 10.0, 0.8 Hz, 1H)	5.97 (dd, J = 9.9, 1.1 Hz, 1H)
3	2.68 (p, J = 7.0 Hz, 1H)	2.71 – 2.63 (m, 1H)	2.67 (tt, J = 8.3, 6.6 Hz, 1H)	2.72 – 2.65 (m, 1H)	2.70 – 2.66 (m, 1H)
4	2.27 – 2.23 (m, 1H)	2.28 – 2.20 (m, 1H)	2.32 – 2.20 (m, 1H)	2.27 – 2.24 (m, 1H)	2.25 (dd, J = 9.4, 5.0 Hz, 1H)
5	2.11 – 2.08 (m, 1H)	2.12 – 2.05 (m, 1H)	2.13 – 2.04 (m, 1H)	2.12 – 2.08 (m, 1H)	2.10 (dd, J = 12.7, 6.0 Hz, 1H)
6	1.99 (d, J = 14.6 Hz, 1H)	1.98 (d, J = 14.5 Hz, 1H)	1.98 (d, J = 14.5 Hz, 1H)	1.99 (d, J = 14.6 Hz, 1H)	1.98 (d, J = 14.2 Hz, 1H)
7	1.71 – 1.56 (m, 6H)	1.72 – 1.54 (m, 6H)	1.71 – 1.53 (m, 6H)	1.73 – 1.47 (m, 7H)	1.68 – 1.48 (m, 7H)
8	1.53 – 1.47 (m, 1H)	1.52 – 1.46 (m, 1H)	1.49 (dddd, J = 12.5, 8.7, 3.7, 1.1 Hz, 1H)	-	-
9	1.39 (d, J = 5.3 Hz, 1H)	1.39 (d, J = 5.3 Hz, 1H)	1.39 (d, J = 5.3 Hz, 1H)	1.40 (d, J = 5.2 Hz, 1H)	1.39 (d, J = 5.0 Hz, 1H)
10	1.28 (s, 3H)	1.28 (s, 3H)	1.27 (s, 3H)	1.29 (s, 3H)	1.28 (s, 3H)
11	1.20 (d, J = 7.3 Hz, 3H)	1.20 (d, J = 7.3 Hz, 3H)	1.20 (d, J = 7.2 Hz, 3H)	1.21 (d, J = 7.2 Hz, 3H)	1.20 (d, J = 7.2 Hz, 3H)
12	1.19 – 1.18 (m, 1H)	1.21 – 1.16 (m, 1H)	1.21 – 1.16 (m, 1H)	1.23 – 1.16 (m, 1H)	1.19 – 1.16 (m, 1H)
13	1.18 (s, 3H)	1.17 (s, 3H)	1.17 (s, 3H)	1.18 (s, 3H)	1.18 (s, 3H)
14	1.06 (s, 3H)	1.05 (s, 3H)	1.05 (s, 3H)	1.06 (s, 3H)	1.06 (s, 3H)
15	0.96 (s, 3H)	0.96 (s, 3H)	0.95 (s, 3H)	0.97 (s, 3H)	0.96 (s, 3H)

Table S2. ^{13}C NMR ($CDCl_3$) Spectroscopic Comparison of Zhai's,[8] Snyder's,[9] Tu's,[10] and natural isolation[11] to our syntheses of Conidiogenone B

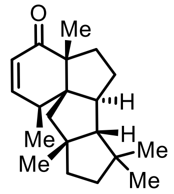

Conidiogenone B

No.	Our Sample (150 MHz)	Zhai' Sample (100 MHz)	Snyder's sample (125 MHz)	Tu's sample (100 MHz)	Natural (125 MHz)
1	206.2	205.9	205.9	206	206
2	154.6	154.4	154.3	154.4	154.5
3	127.6	127.4	127.5	127.4	127.4
4	73.8	73.7	73.7	73.6	73.6
5	59.1	59	59	58.9	58.9
6	57.6	57.4	57.4	57.4	57.4
7	56.2	56	56	56	56
8	52.4	52.3	52.3	52.3	52.3
9	48.1	48	48	48	48
10	42.9	42.7	42.7	42.7	42.7
11	40.9	40.8	40.8	40.7	40.7
12	39.2	39	39	39	39
13	39.1	38.9	38.9	38.9	38.9
14	37.7	37.5	37.5	37.5	37.5
15	34.5	34.3	34.4	34.3	34.3
16	32.7	32.5	32.5	32.5	32.5
17	31.1	31	31	31	31
18	27.3	27.1	27.1	27.1	27.1
19	27.1	21	21	21	21
20	18.9	18.8	18.9	18.8	18.8

E. References

1. Trost, B. M.; Bream, R. N.; Xu, J. Asymmetric Allylic Alkylation of Cyclic Vinylogous Esters and Thioesters by Pd-Catalyzed Decarboxylation of Enol Carbonate and β-Ketoester Substrates. *Angew. Chem., Int. Ed.* **2006**, 45, 3109–3112.
2. Lo, J. C.; Yabe, Y.; Baran, P. S. A Practical and Catalytic Reductive Olefin Coupling. *J. Am. Chem. Soc.* **2014**, 136, 1304–1307.
3. Tanyeli, C.; Özdemirhan; D. Mn(III) acetate-mediated regioselective benzylation of various α,β-unsaturated and β-alkoxy-α,β-unsaturated ketones. *Tetrahedron Lett.* **2003**, 44, 7311–7313.
4. Kawamoto, Y.; Ozone, D.; Kobayashi, T.; Ito, H. Enantioselective Total Synthesis of Chondrosterins I and J by Catalytic Asymmetric Intramolecular Aldol Reaction Using Chiral Diamine Catalyst. *Eur. J. Org. Chem.* **2020**, 2020, 4050–4058.
5. Chen, Z.-T.; Xiao, T.; Tang, P.; Zhang, D.; Qin, Y. Total synthesis of akuammiline alkaloid (+)-strictamine. *Tetrahedron* **2018**, 74, 1129–1134.
6. Turner, O. J.; Murphy, J. A.; Hirst, D. J.; Talbot, E. P. A. Hydrogen Atom Transfer-Mediated Cyclisations of Nitriles. *Chem. - Eur. J.* **2018**, 24, 18658–18662.
7. Liu, J.; Ma, D. A Unified Approach for the Assembly of Atisine- and Hetidine-type Diterpenoid Alkaloids: Total Syntheses of Azitine and the Proposed Structure of Navirine C. *Angew. Chem., Int. Ed.* **2018**, 57, 6676–6680.
8. Xu, B.; Xun, W.; Su, S.; Zhai, H. Total Syntheses of (−)-Conidiogenone B, (−)-Conidiogenone, and (−)-Conidiogenol. *Angew. Chem., Int. Ed.* **2020**, *59*, 16475–16479.
9. Hou, S.-H.; Tu, Y.-Q.; Wang, S.-H.; Xi, C.-C.; Zhang, F.-M.; Wang, S.-H.; Li, Y.-T.; Liu, L. Total Syntheses of the Tetracyclic Cyclopiane Diterpenes Conidiogenone, Conidiogenol, and Conidiogenone B. *Angew. Chem., Int. Ed.* **2016**, *55*, 4456–4460.
10. Hu, P.; Chi, H. M.; DeBacker, K. C.; Gong, X.; Keim, J. H.; Hsu, I. T.; Snyder, S. A. Quaternary-Centre-Guided Synthesis of Complex Polycyclic Terpenes. *Nature* **2019**, *569*, 703–707.
11. Du, L.; Li, D.; Zhu, T.; Cai, S.; Wang, F.; Xiao, X.; Gu, Q. New Alkaloids and Diterpenes from a Deep Ocean Sediment Derived Fungus *Penicillium* Sp. *Tetrahedron* **2009**, *65*, 1033–1039.

F. ^{1}H and ^{13}C NMR spectra.

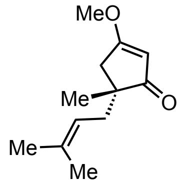

38

^{1}H NMR (400 MHz, $CDCl_3$)

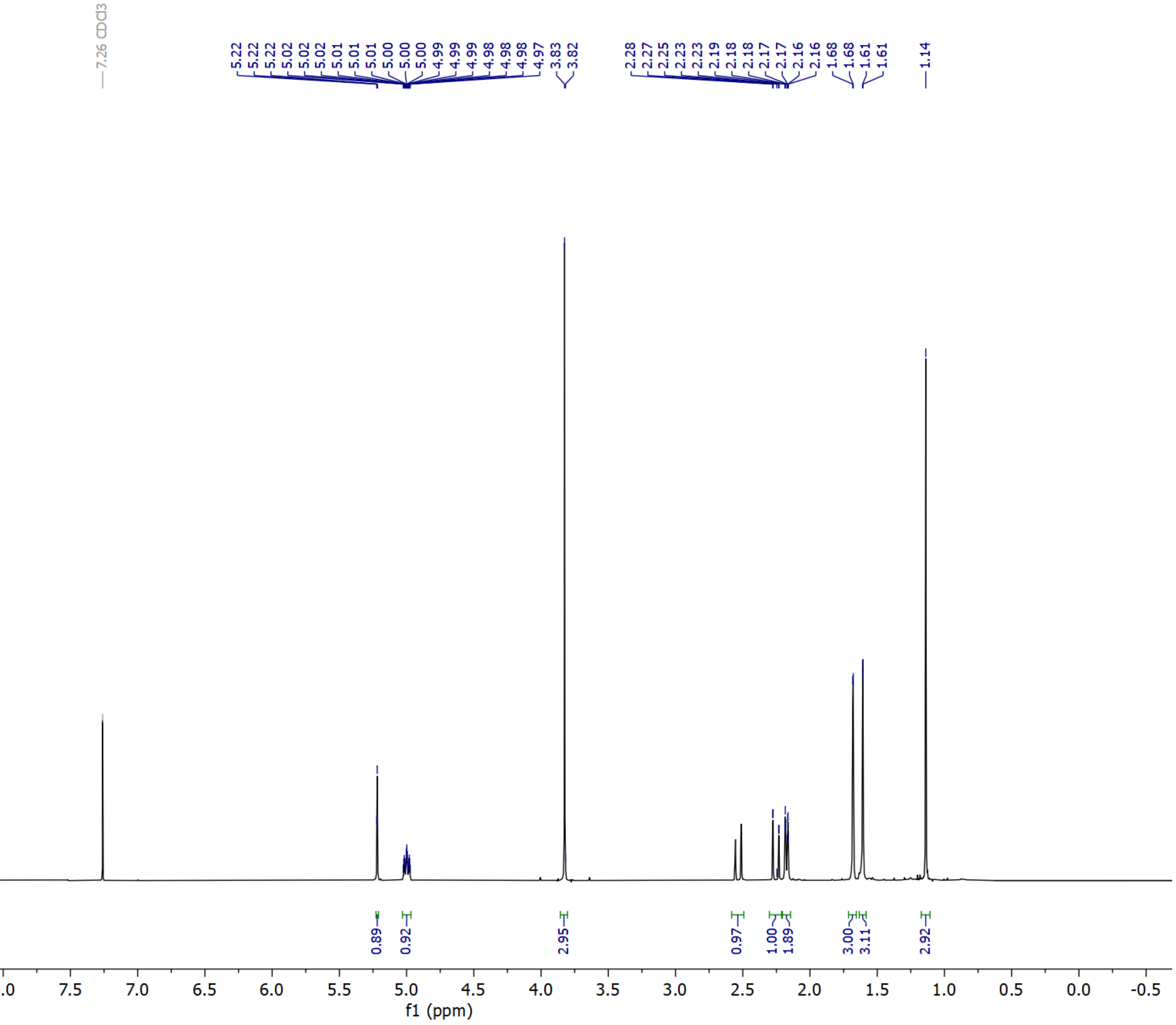

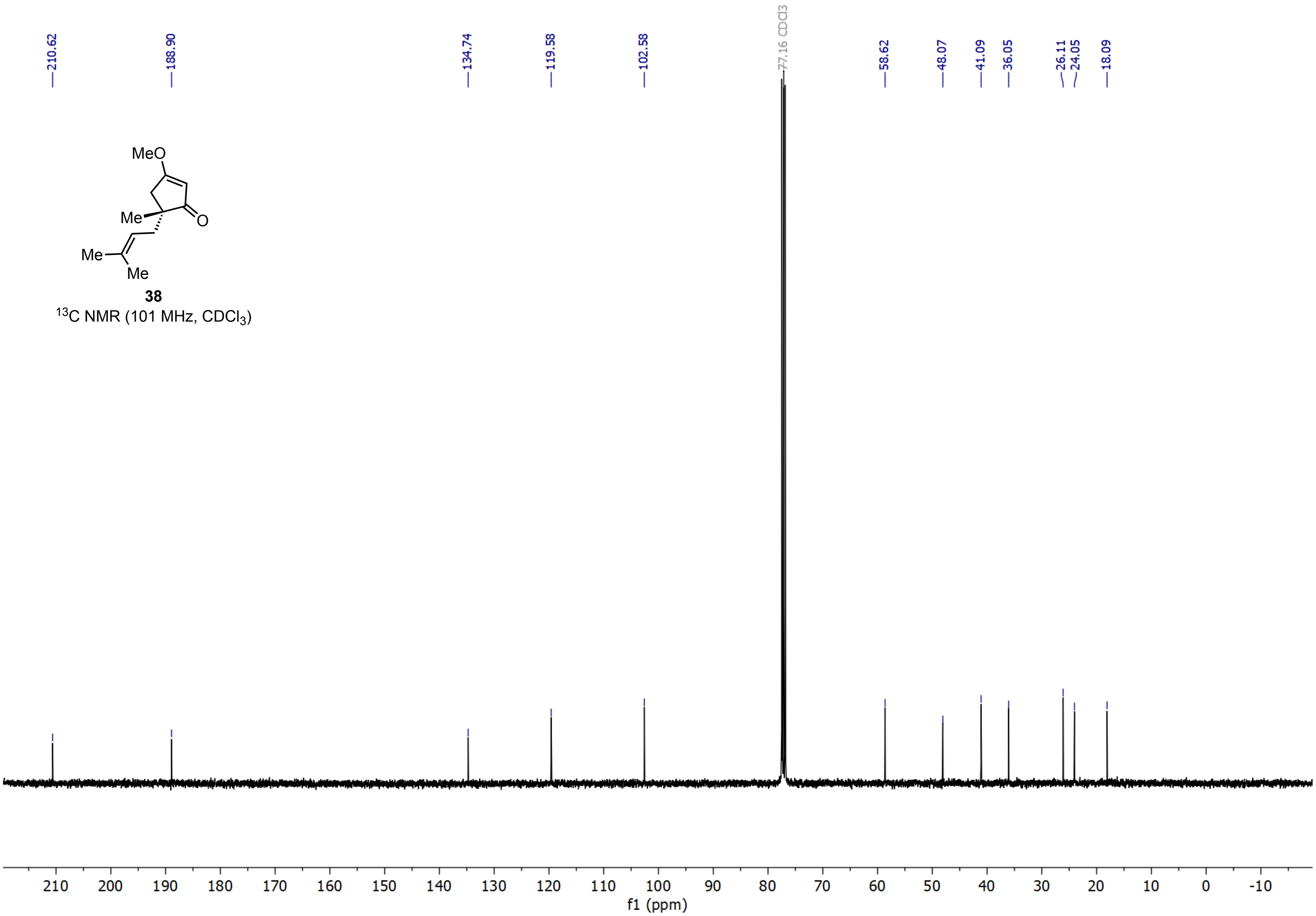

38

^{13}C NMR (101 MHz, $CDCl_3$)

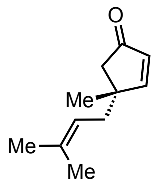

39

^{1}H NMR (400 MHz, $CDCl_3$)

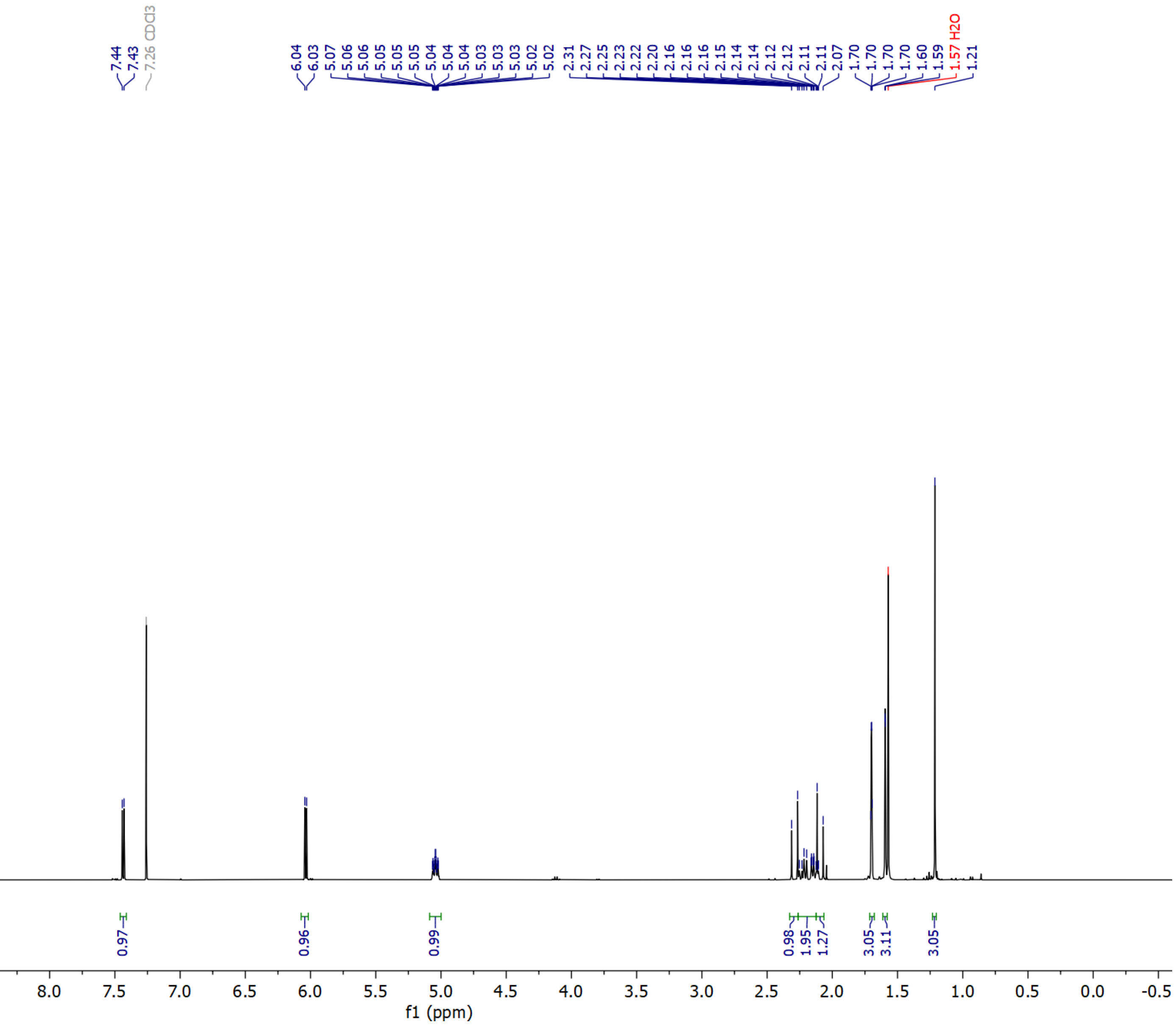

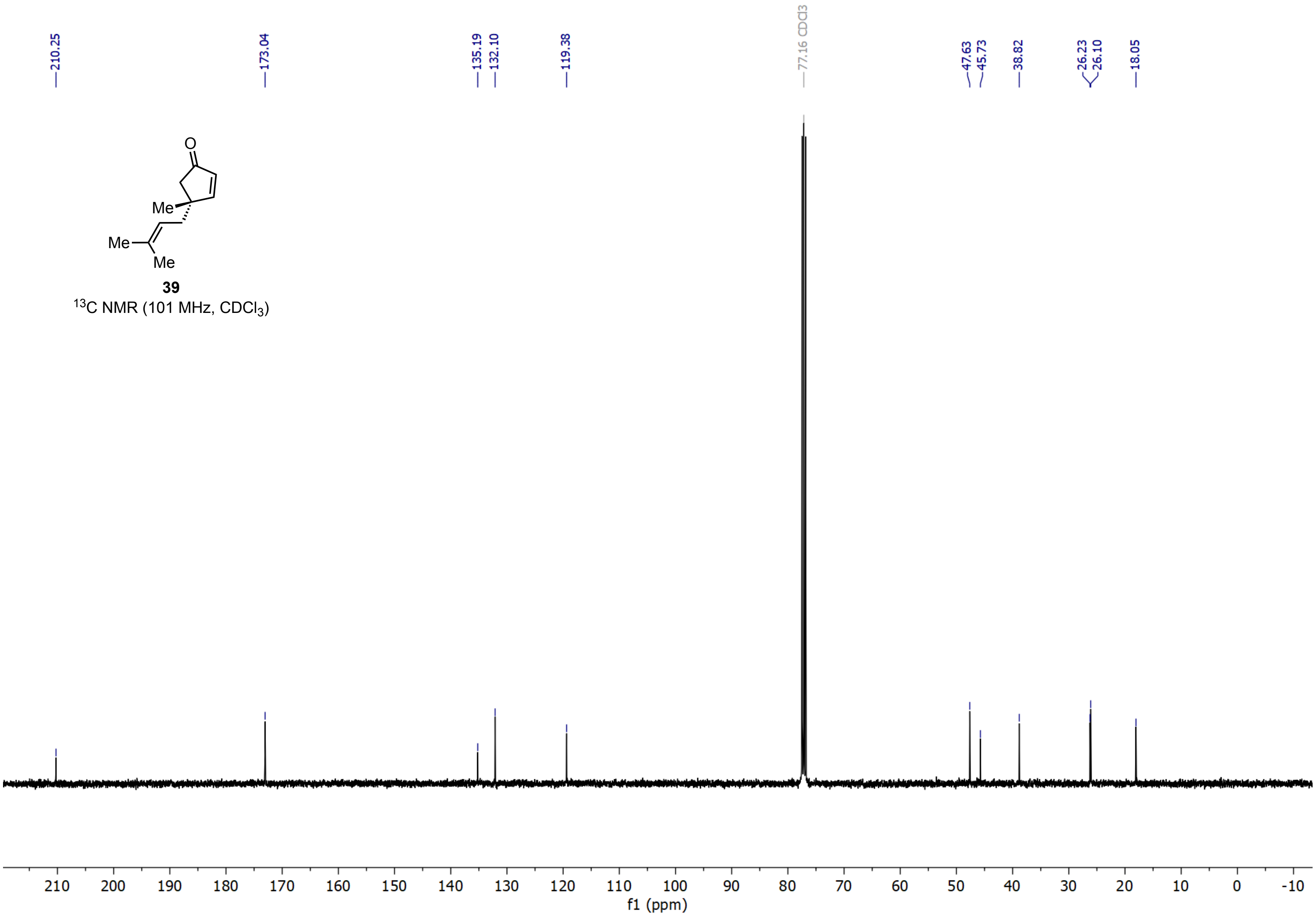

210.25
173.04
135.19
132.10
119.38
77.16 CDCl3
47.63
45.73
38.82
26.23
26.10
18.05
O
Me
Me
Me
39
^{13}C NMR (101 MHz, $CDCl_3$)
210
200
190
180
170
160
150
140
130
120
110
100
90
80
70
60
50
40
30
20
10
0
-10
f1 (ppm)

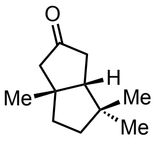

33

^{1}H NMR (400 MHz, $CDCl_3$)

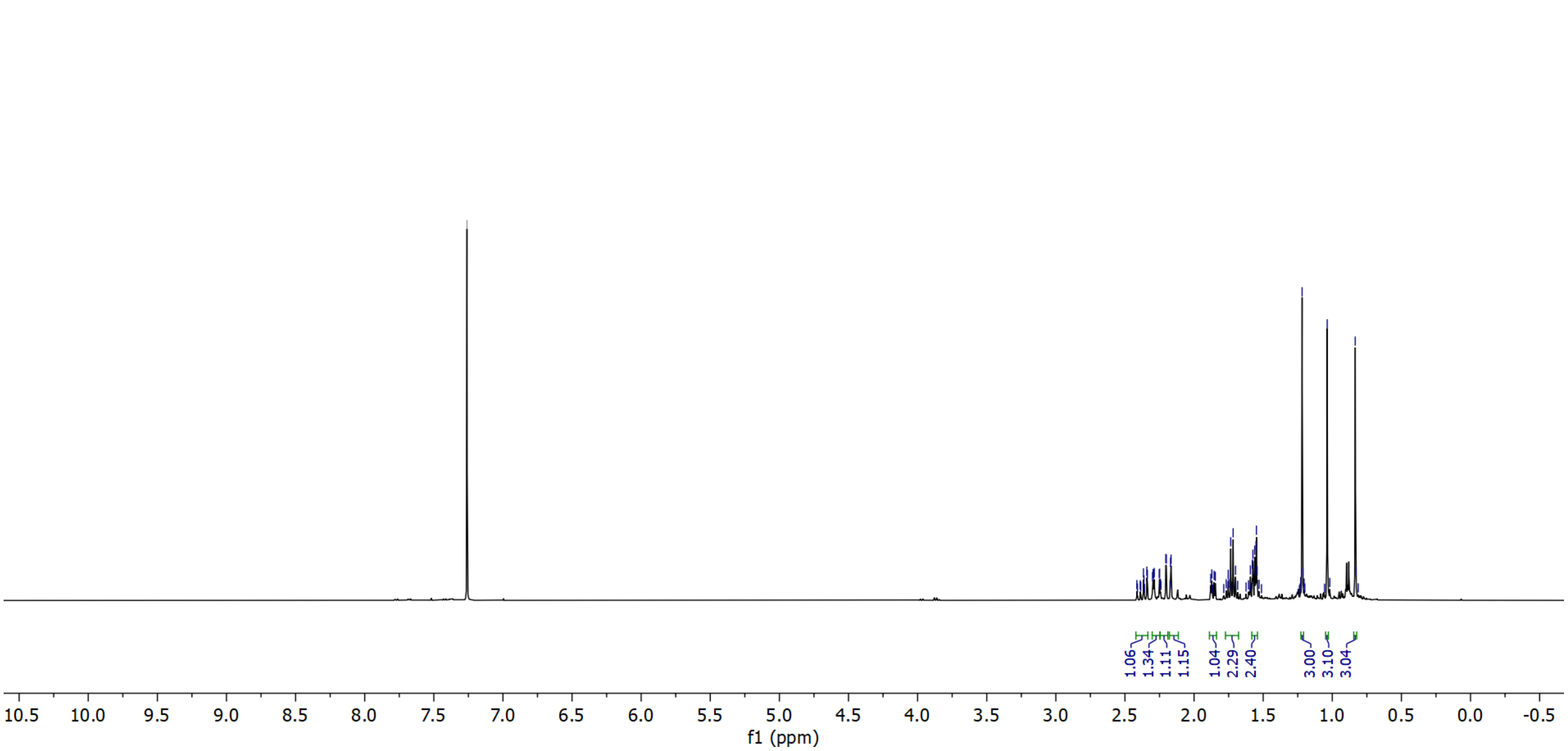

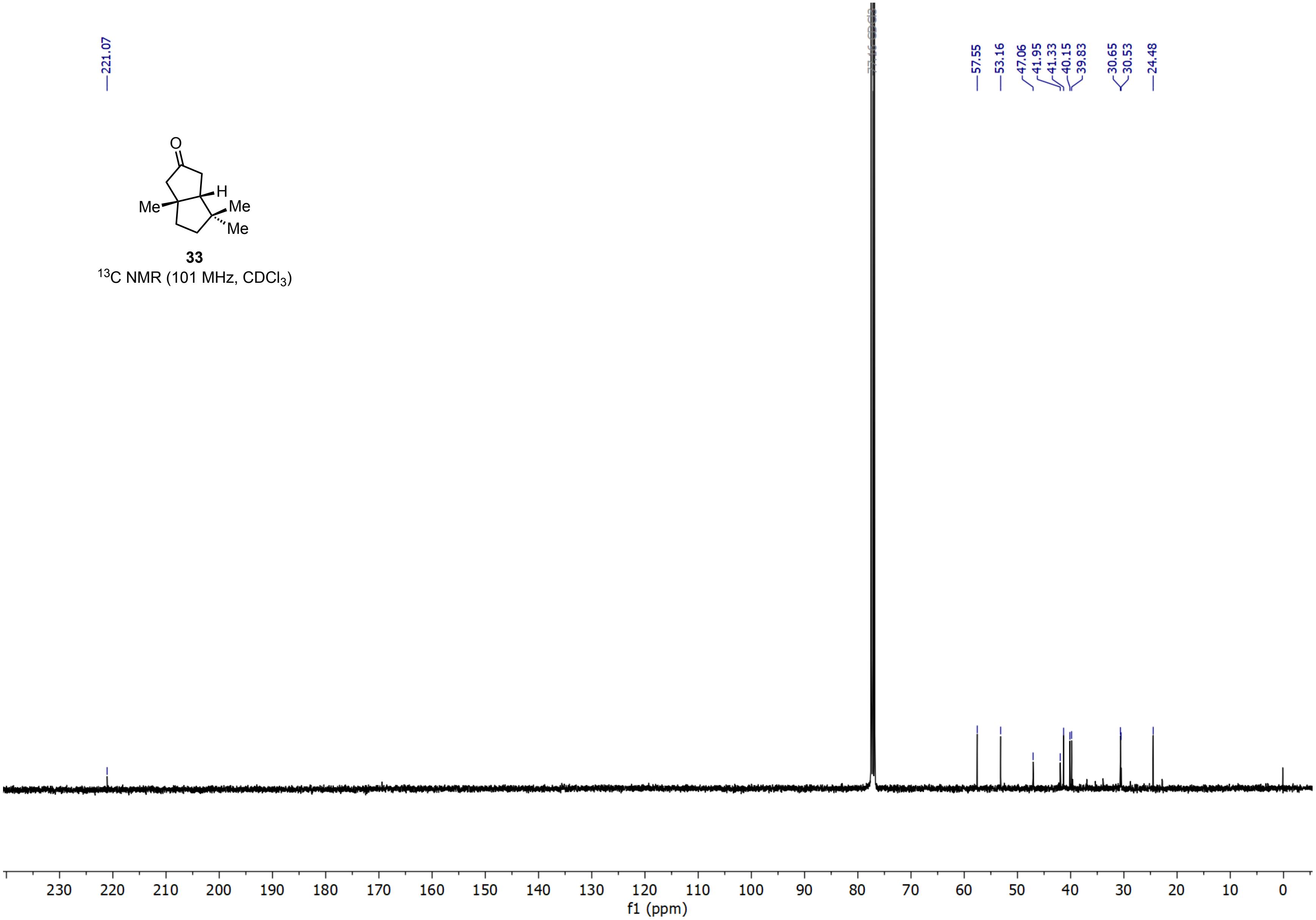

33

^{13}C NMR (101 MHz, $CDCl_3$)

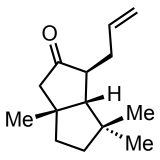

40

^{1}H NMR (400 MHz, $CDCl_3$)

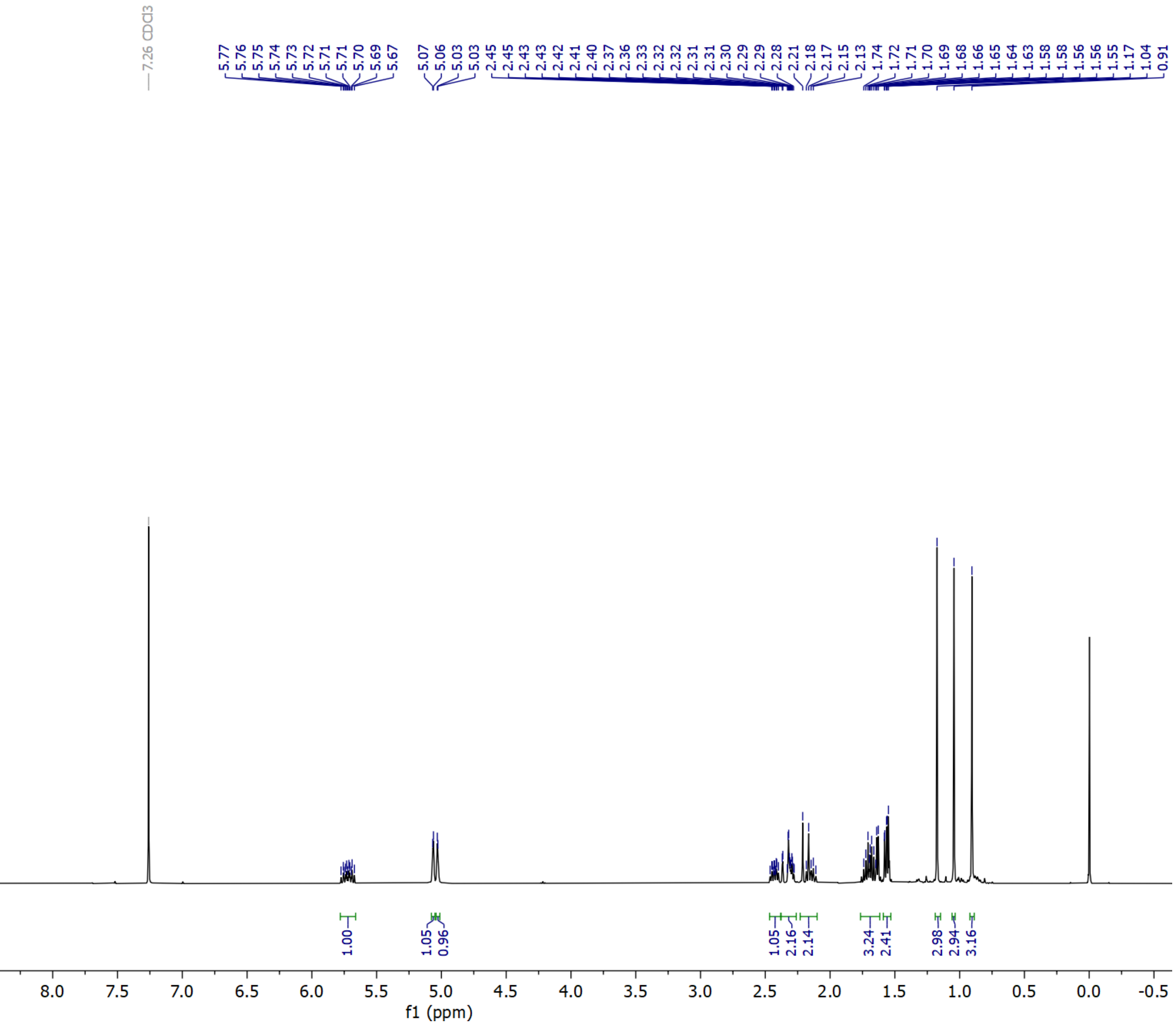

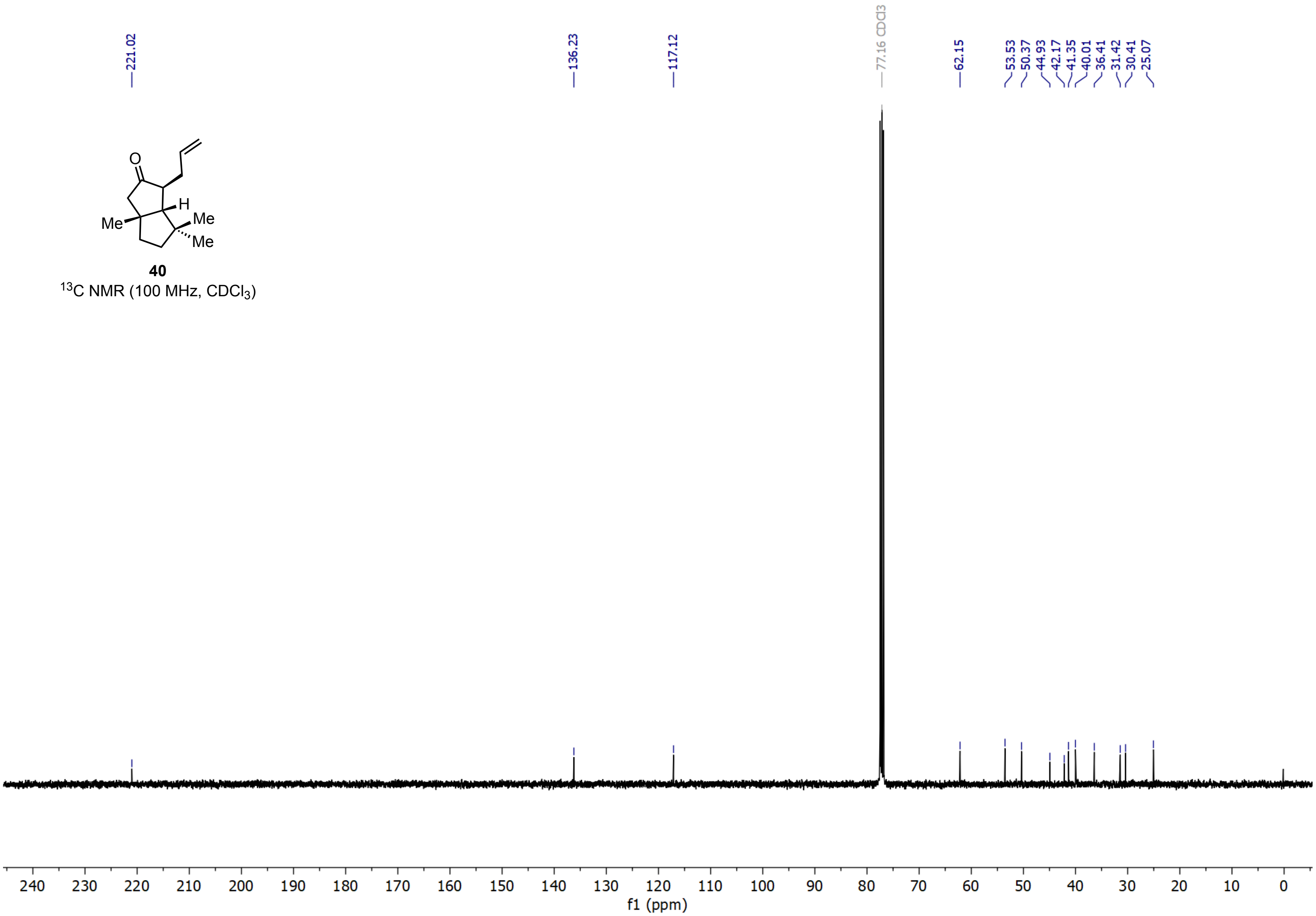

40

^{13}C NMR (100 MHz, $CDCl_3$)

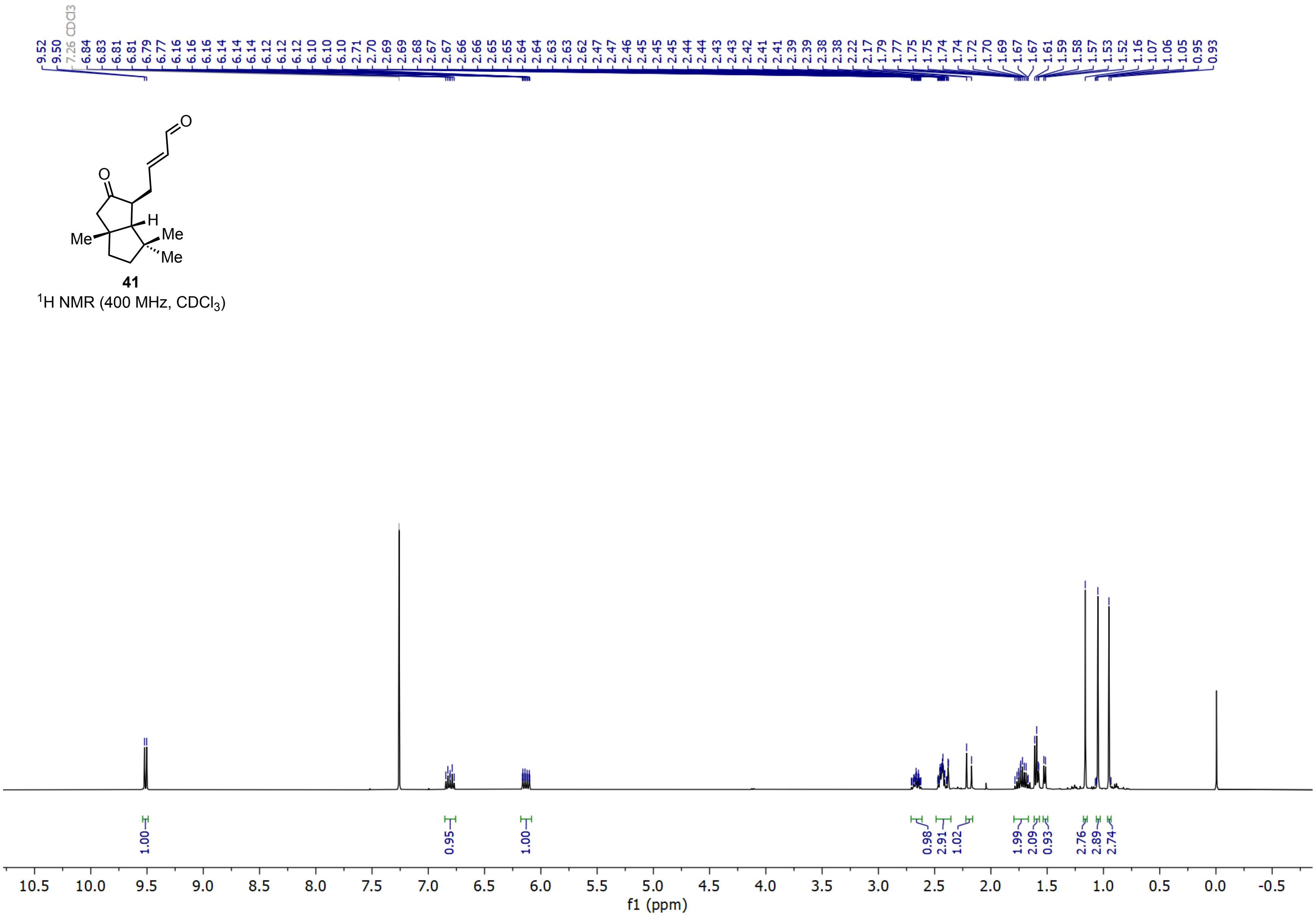
9.52 9.50 7.26 CDCl3 6.84 6.83 6.81 6.81 6.79 6.77 6.16 6.16 6.16 6.14 6.14 6.12 6.12 6.12 6.10 6.10 6.10 2.71 2.70 2.69 2.69 2.68 2.67 2.67 2.66 2.66 2.65 2.65 2.64 2.64 2.63 2.63 2.62 2.47 2.47 2.46 2.45 2.45 2.45 2.44 2.44 2.43 2.43 2.42 2.41 2.41 2.39 2.39 2.38 2.38 2.22 2.17 1.79 1.77 1.75 1.75 1.74 1.74 1.72 1.70 1.69 1.67 1.67 1.61 1.59 1.58 1.57 1.53 1.52 1.16 1.07 1.06 1.05 0.95 0.93
O
O
H
Me
Me
Me
41
^{1}H NMR (400 MHz, $CDCl_3$)
1.00
0.95
1.00
0.98
2.91
1.02
1.99
2.09
0.93
2.76
2.89
2.74
10.5 10.0 9.5 9.0 8.5 8.0 7.5 7.0 6.5 6.0 5.5 5.0 4.5 4.0 3.5 3.0 2.5 2.0 1.5 1.0 0.5 0.0 -0.5
f1 (ppm)

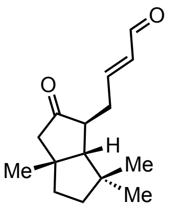

41

^{13}C NMR (101 MHz, $CDCl_3$)

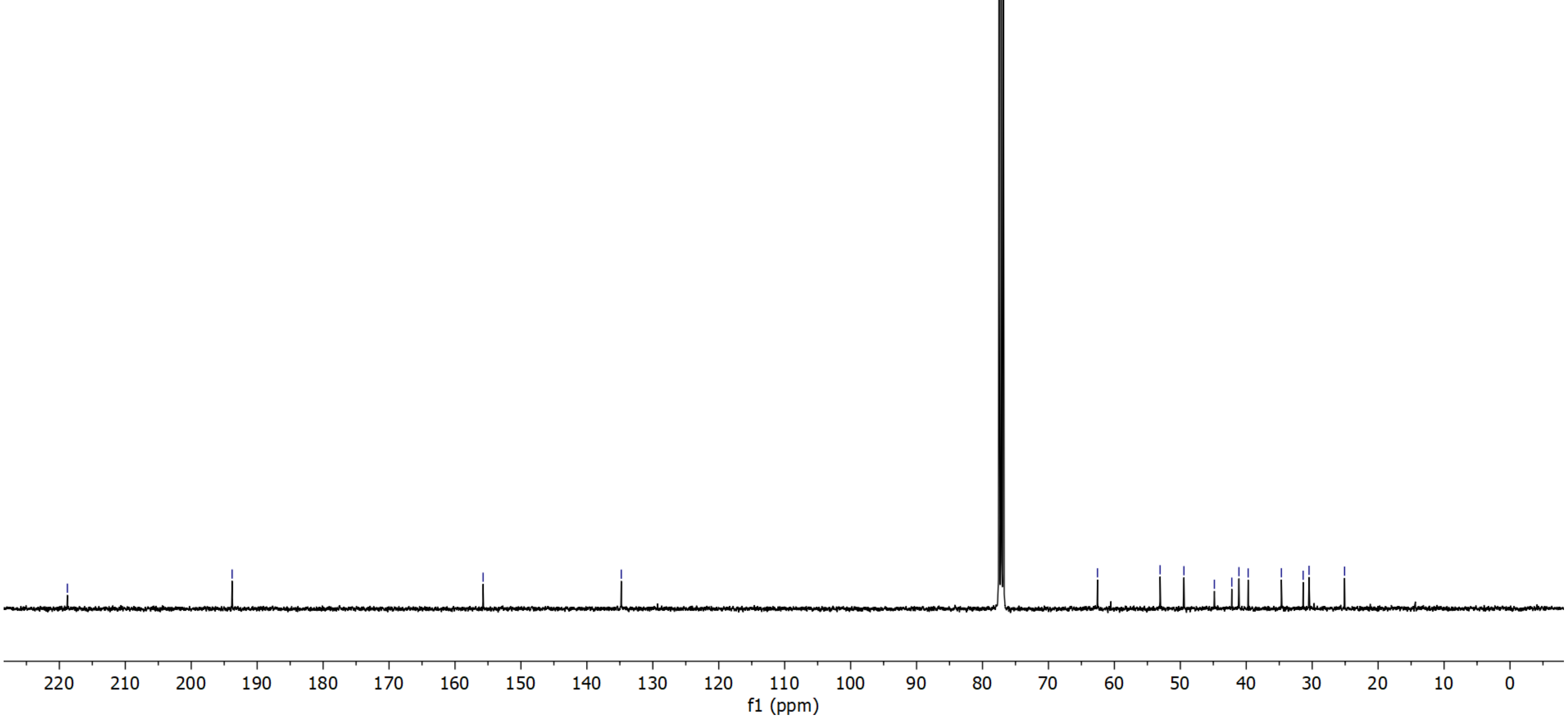

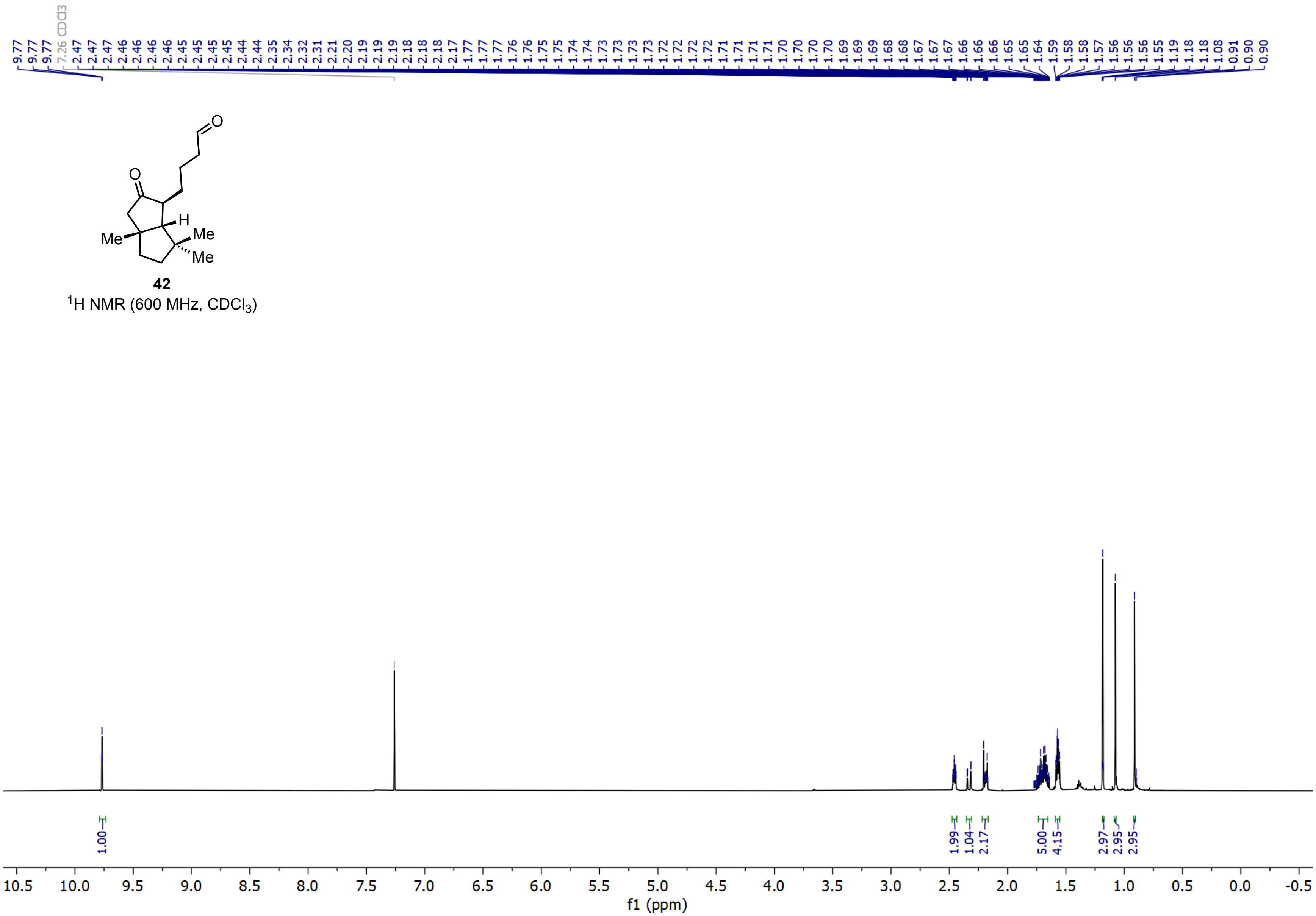

42

^1H NMR (600 MHz, $CDCl_3$)

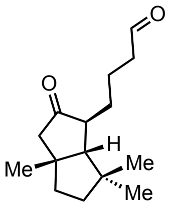

42

^{13}C NMR (101 MHz, $CDCl_3$)

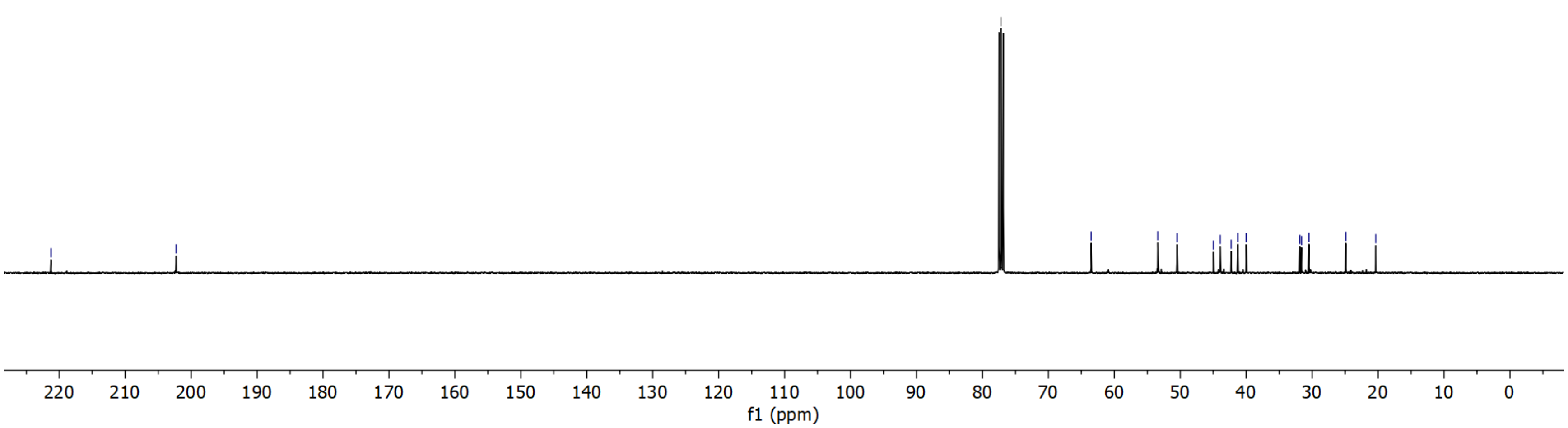

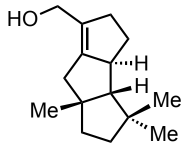

32

^{1}H NMR (400 MHz, $CDCl_3$)

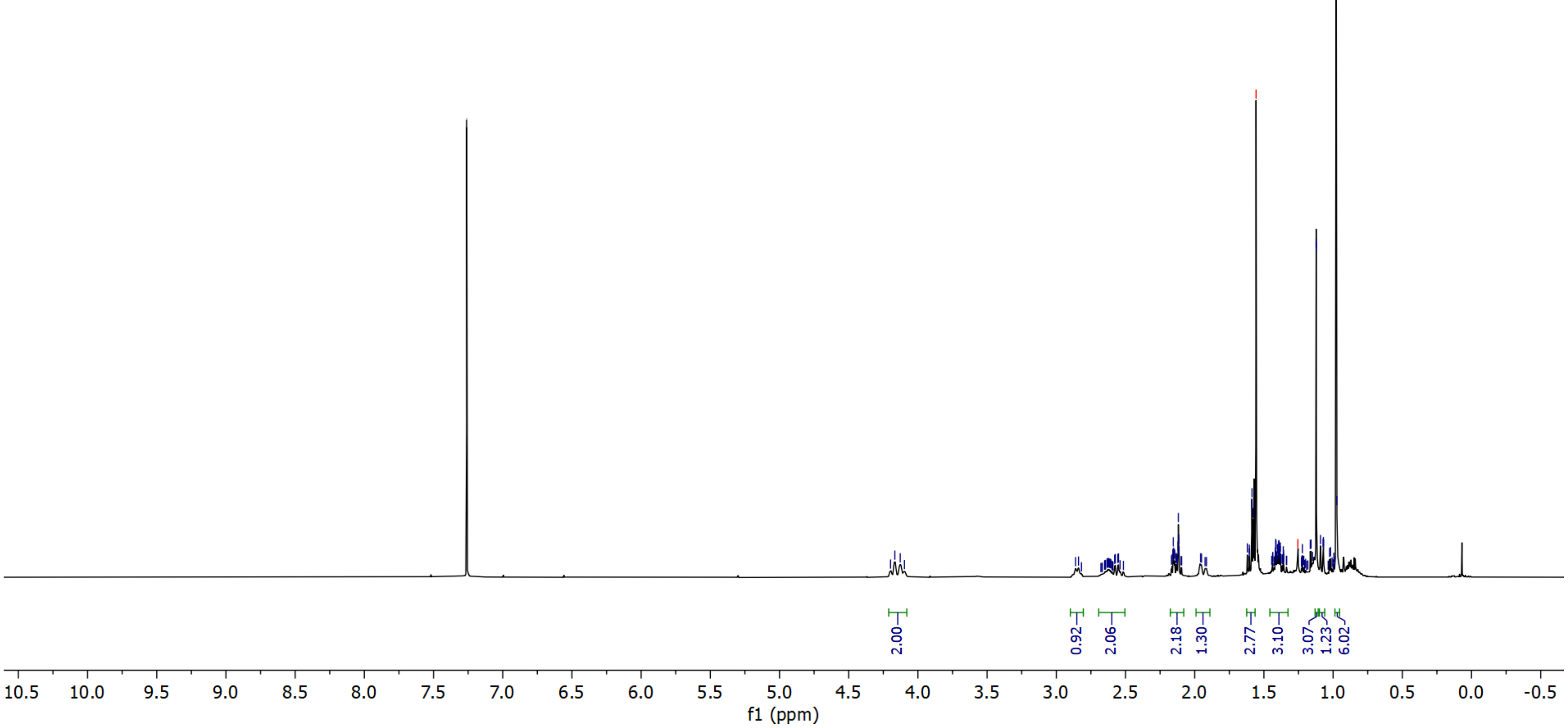

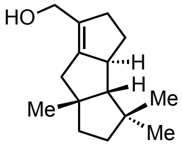

32

^{13}C NMR (101 MHz, $CDCl_3$)

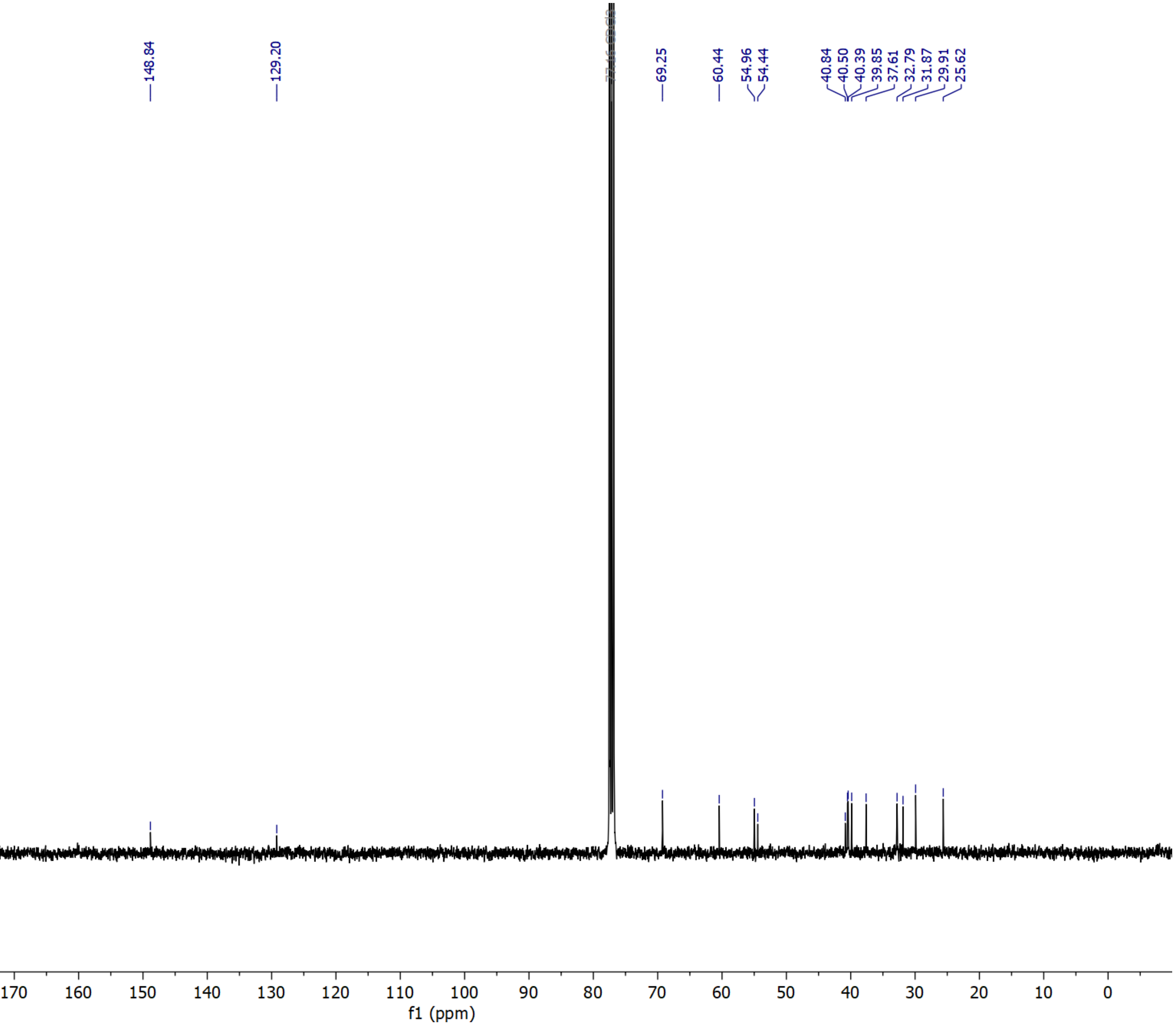

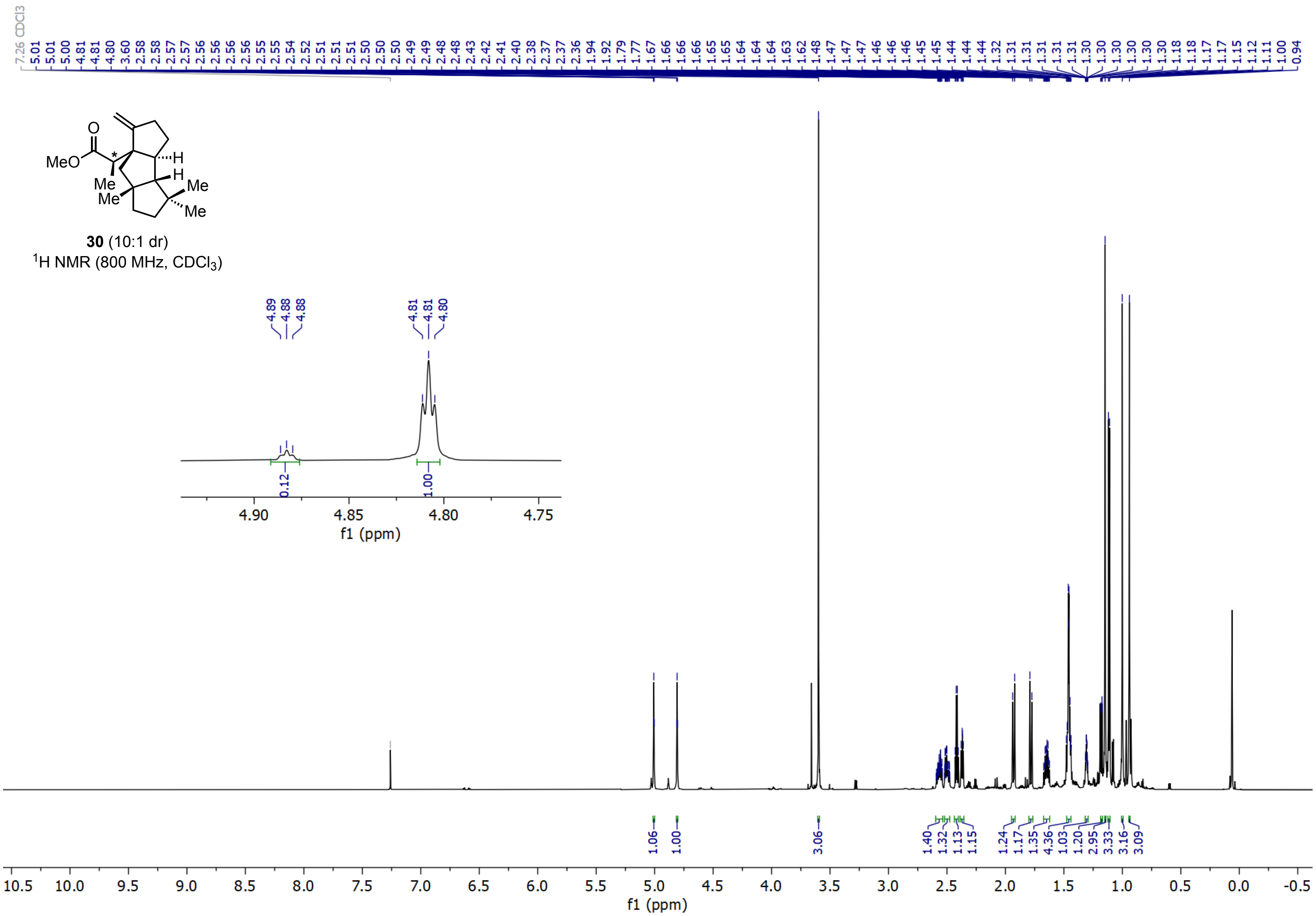
MeO
O
Me
Me
H
H
Me
Me
30 (10:1 dr)
1H NMR (800 MHz, CDCl3)
4.90
4.85
4.80
4.75
f1 (ppm)
f1 (ppm)

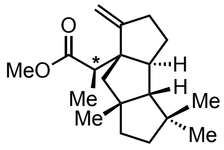

30 (10:1 dr)
^{13}C NMR (201 MHz, $CDCl_3$)

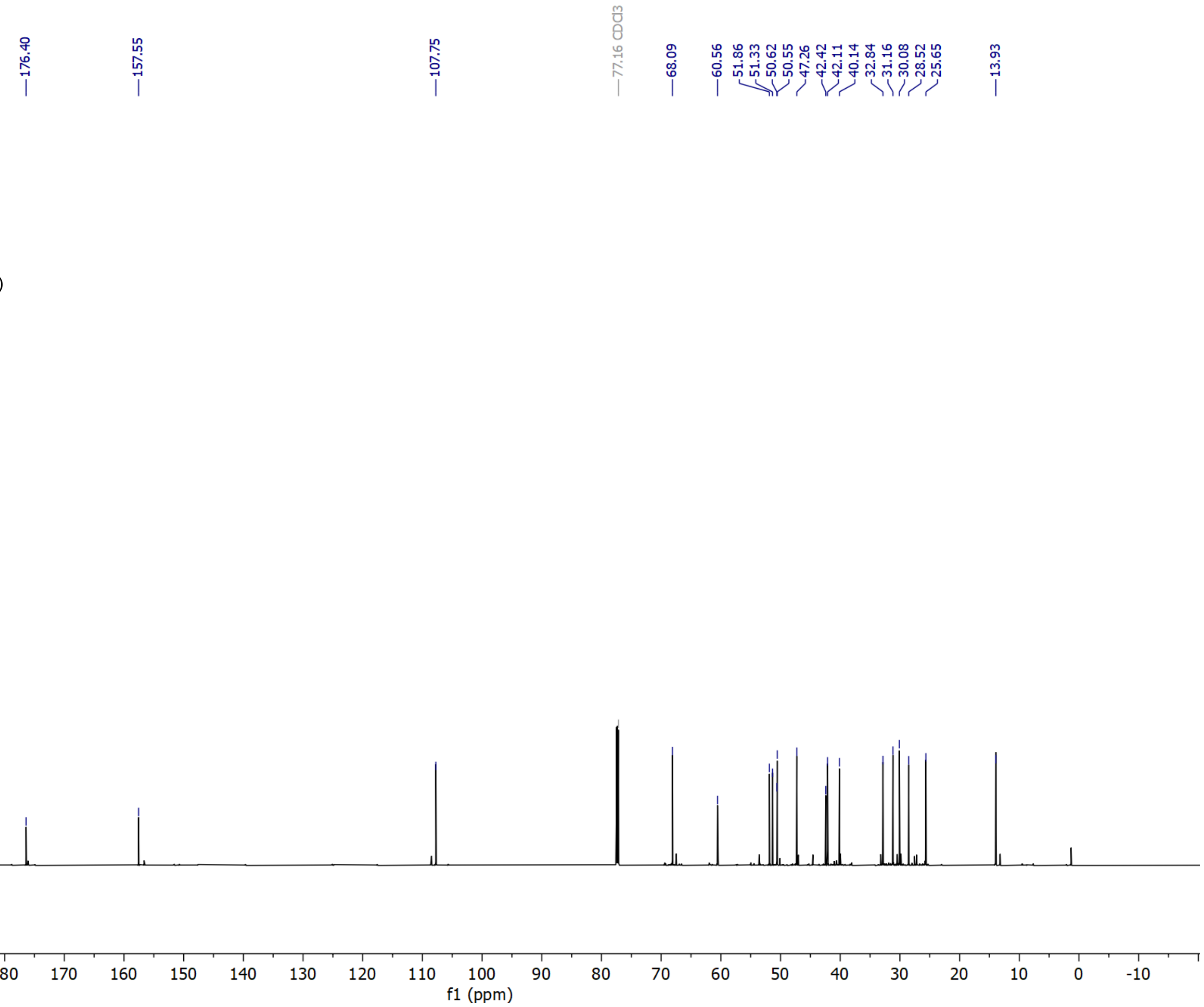

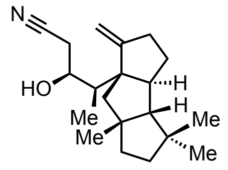

29 (10:1 dr)
^{1}H NMR (800 MHz, $CDCl_3$)

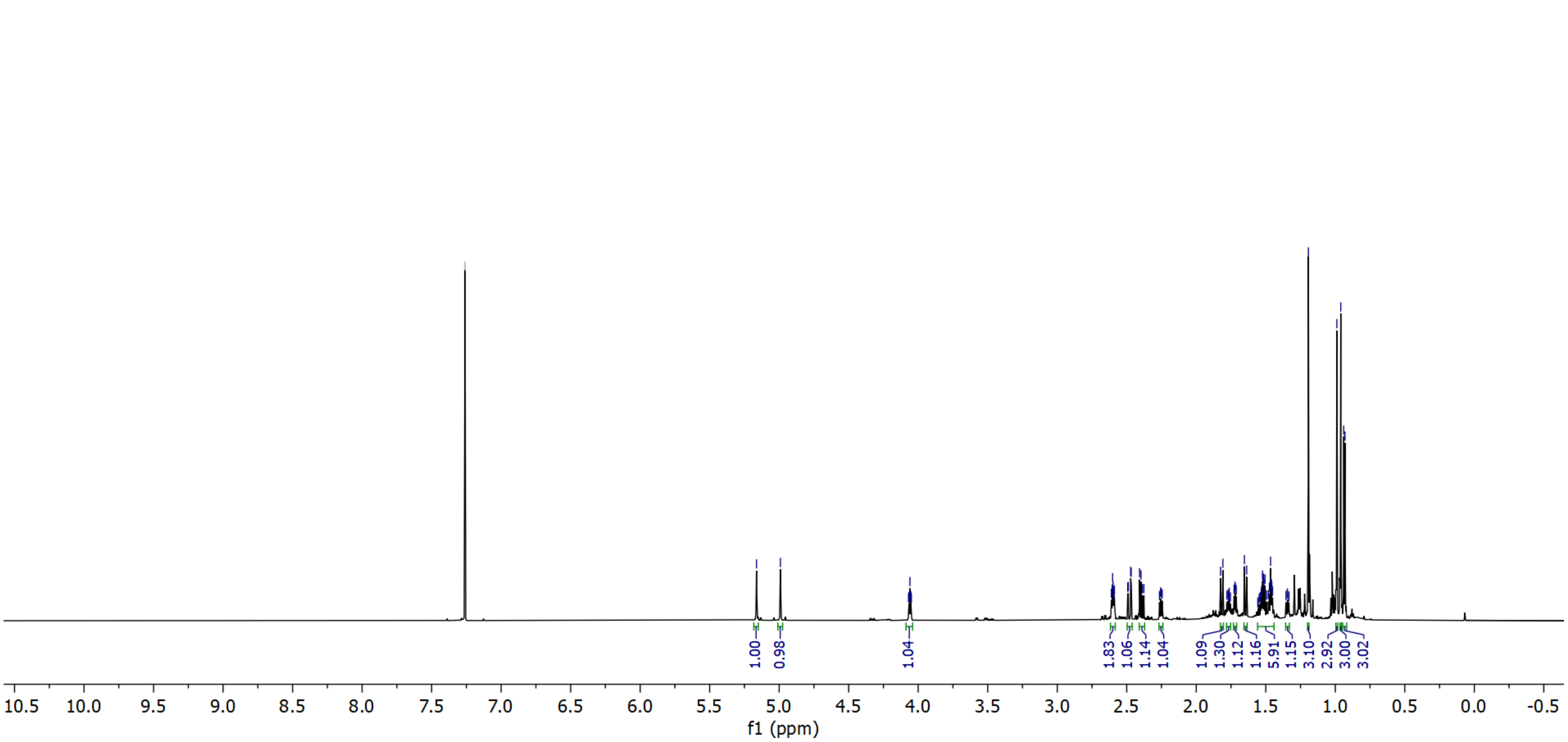

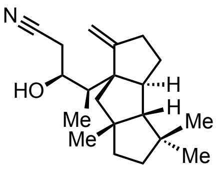

29 (10:1 dr)
^{13}C NMR (101 MHz, $CDCl_3$)

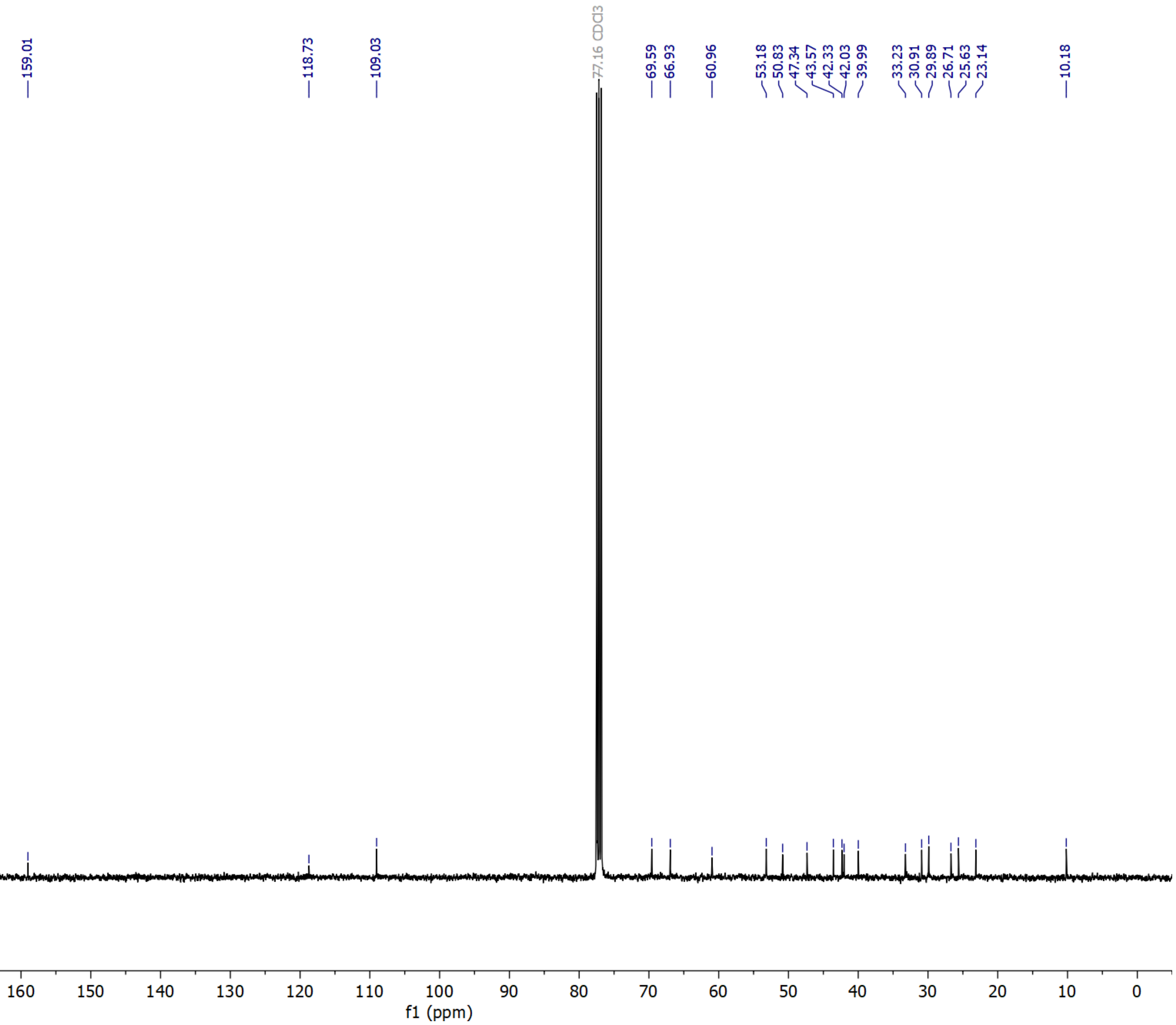

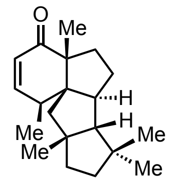

Conidiogenone B (**2**)
^{1}H NMR (800 MHz, $CDCl_3$)

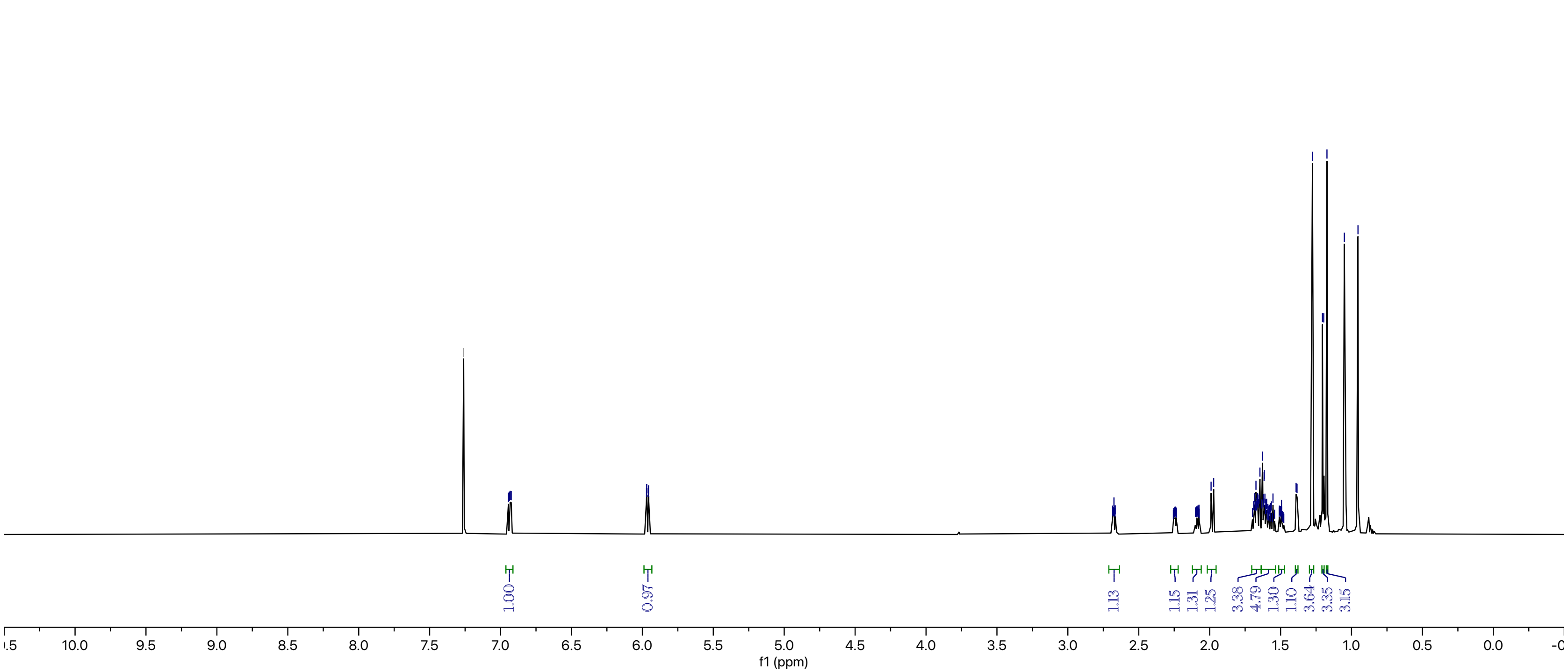

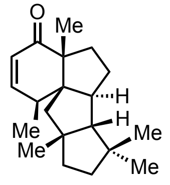

Conidiogenone B (**2**)
^{13}C NMR (151 MHz, $CDCl_3$)

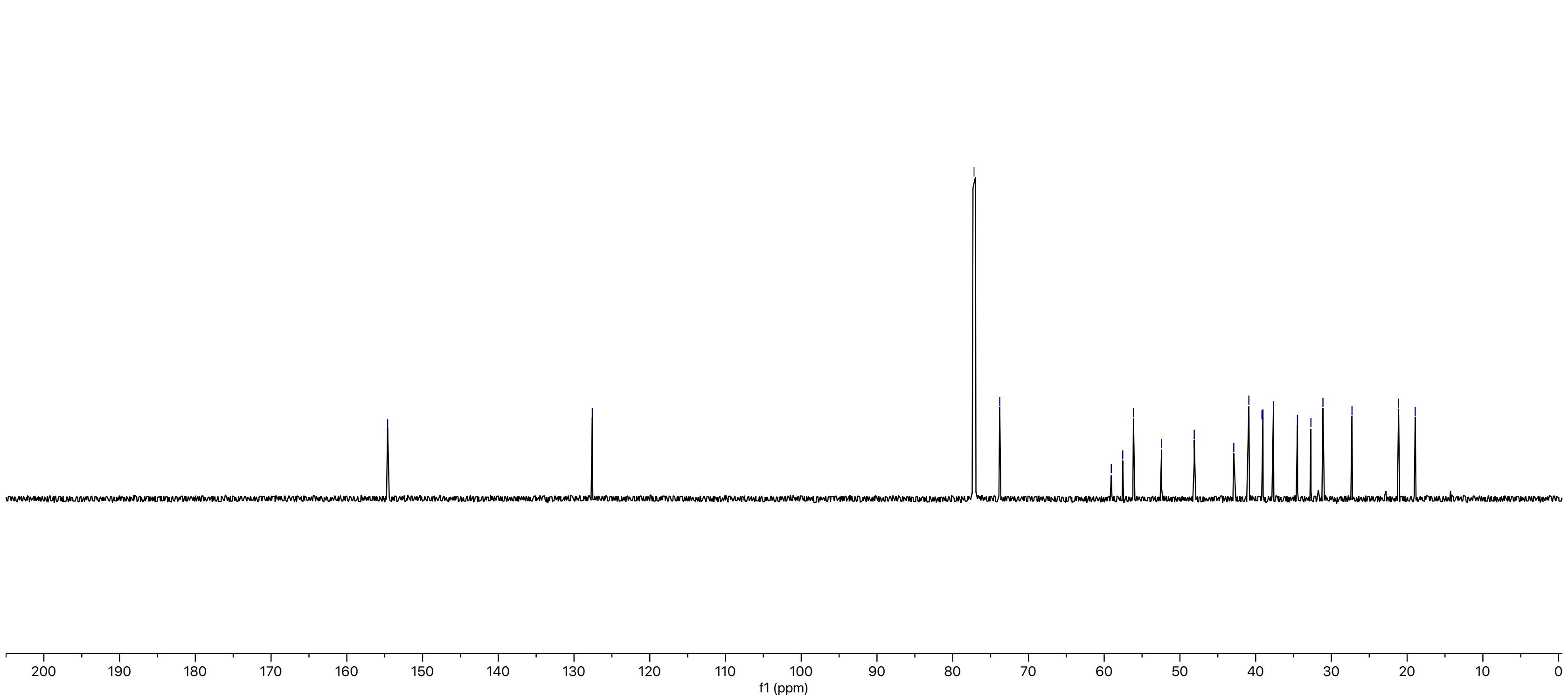

Supplement: Supplementary file 1 [file ja6c02334_si_001.pdf]
